# Socio‐Technical Risk‐Informed AI‐Driven Automation Trustworthiness Evaluation (ST‐RATE): Part 1‐Conceptualization

**DOI:** 10.1111/risa.70367

**Published:** 2026-10-07

**Authors:** Muhammad Hammad Khalid, Ha Bui, Ahmad Al Rashdan, Zahra Mohaghegh

**Affiliations:** ^1^ Socio‐Technical Risk Analysis (SoTeRiA) Laboratory Urbana Illinois USA; ^2^ Department of Nuclear, Plasma, and Radiological Engineering University of Illinois at Urbana‐ Champaign Urbana Illinois USA; ^3^ Idaho National Laboratory (INL) Idaho Falls Idaho USA

**Keywords:** AI‐driven automation trustworthiness, artificial intelligence (AI), nuclear power plants, risk‐informed, socio‐technical, uncertainty analysis

## Abstract

The growing integration of artificial intelligence (AI)‐driven technologies into nuclear power plant (NPP) operations and maintenance holds significant potential to enhance safety, operational efficiency, and reliability. However, deploying such AI‐driven automation technologies in high‐consequence settings requires a rigorous, traceable, and risk‐informed evaluation of their trustworthiness. Although researchers across various domains have proposed definitions and metrics for AI‐driven automation trustworthiness, no agreed‐upon formal or quantitative method exists that is suitable for the highly regulated nuclear sector. To address this gap, this paper, the first in a two‐part series, presents three contributions: (I) a cross‐disciplinary literature review that categorizes existing definitions and evaluation methodologies for AI‐driven automation trustworthiness and identifies their inherent limitations with respect to applicability in the nuclear domain; (II) develops a new definition of AI‐driven automation trustworthiness as “the degree of confidence that the AI‐driven automation system will function as expected across its conditions of use” to address the limitations identified in Contribution I; and (III) building on the new definition, conceptualizes the Socio‐Technical Risk‐informed AI‐driven Automation Trustworthiness Evaluation (ST‐RATE) methodology, grounded in three foundational pillars: (a) risk‐informed performance expectations, (b) socio‐technical context across operational conditions, and (c) uncertainty‐based trustworthiness evaluation. While developed primarily for the nuclear domain, the definition and ST‐RATE methodology has the potential to be adapted for other safety‐critical domains with appropriate modifications. The ST‐RATE methodological framework is operationalized and demonstrated through an AI‐driven automated firewatch case study in a companion paper (Companion paper Part 2, 2025). ST‐RATE is the first‐of‐its‐kind methodology that enables risk‐informed and uncertainty‐based quantification of trustworthiness despite limited empirical data and without over‐reliance on subjective expert judgment, addressing the key limitations of existing approaches identified in the literature review.

## 1 Introduction and Statement of Objectives

In its pursuit of enhanced operational efficiency and safety, the US nuclear industry is advancing plant modernization initiatives centered on the integration of AI‐driven automation technologies (Hall et al. 2024; Lin et al. 2025; Mortenson et al. 2024). While anticipated benefits include predictive diagnostics, autonomous control, and decision support, these technologies also introduce complex challenges including developing strong business cases for adoption (Kovesdi et al. 2021), ensuring trustworthiness of AI‐driven automation systems (Krarup et al. 2023; Saboury et al. 2023), improving transparency (Khalid et al. 2026; Skraaning Jr et al. 2020; van de Merwe et al. 2022), and addressing regulatory and licensing burdens (Muhlheim et al. 2023).

Before committing to AI‐driven automation in safety‐critical applications, decision‐makers require rigorous, risk‐informed evidence that the system will be sufficiently trustworthy within its intended conditions of use (Al Rashdan et al. 2021; S. G. Kim et al. 2025; Kollias et al. 2022). However, the existing literature points to two overarching limitations: (1) a lack of cross‐domain consensus on the definition of AI‐driven automation trustworthiness and the absence of a widely accepted evaluation framework (Roszel 2024); and (2) existing evaluation approaches rely heavily on qualitative or semi‐quantitative methods and empirical validation, both of which are problematic in the nuclear context. Qualitative approaches raise subjectivity concerns that complicate regulatory review, while empirical validation is constrained by: (i) limited availability of AI‐driven automation output data; (ii) multiple sources of input‐level uncertainty within the AI‐driven automation system hierarchy; and (iii) potential mismatch between available data and the specific operational conditions of intended deployment.

To address these challenges, this research presents a two‐part paper series that conceptualizes and develops a novel framework for evaluating AI‐driven automation trustworthiness, termed the Socio‐Technical Risk‐informed AI‐driven Automation Trustworthiness Evaluation (ST‐RATE). This paper (Part 1) establishes the conceptual foundations through three contributions:

- A cross‐disciplinary literature review that categorizes existing definitions and evaluation methodologies for AI‐driven automation trustworthiness and assesses their limitations for nuclear domain applicability (Section 3);
- A new definition of AI‐driven automation trustworthiness as “the degree of confidence that the AI‐driven automation system will function as expected across its conditions of use,” formulated to address the limitations identified in contribution I (Section 4); and
- The conceptualization of ST‐RATE, grounded in three foundational pillars: risk‐informed performance expectations, socio‐technical context across operational conditions, and uncertainty‐based trustworthiness evaluation, that together enable rigorous, quantitative trustworthiness evaluation despite limited empirical data and without over‐reliance on subjective expert judgment (Section 4).

To the best of the authors' knowledge, ST‐RATE is the first‐of‐its‐kind methodology that quantifies AI‐driven automation trustworthiness through a unified, risk‐informed, and uncertainty‐based framework explicitly modeling socio‐technical interactions across the full spectrum of operational conditions. While developed primarily for the nuclear domain, it has the potential to be adapted to other safety‐critical domains with appropriate modifications. The companion paper (Khalid et al. 2026) operationalizes the three pillars into a methodological and computational framework and demonstrates their application through an AI‐driven automated firewatch (AI‐AFW) case study in an NPP setting.

Section 2 defines the scope of AI‐driven automation systems considered in this research for trustworthiness evaluation. Section 3 reviews existing definitions and evaluation methodologies of AI‐driven automation trustworthiness and highlights their limitations for nuclear applications. The identified gaps motivate the development of a new definition and the conceptualization of the ST‐RATE methodology in Section 4.

## 2 AI‐Driven Automation in Nuclear Power Plants Operations and Maintenance (O&M)

This section establishes the application domain and conceptual scope underpinning the literature review in Section 3 and the ST‐RATE conceptualization in Section 4. Sub‐section 2.1 summarizes the key prospects and challenges associated with AI‐driven automation in NPP Operations and Maintenance (O&M), motivating the need for a rigorous trustworthiness evaluation framework. Sub‐section 2.2 defines the scope of AI‐driven automation systems through a pragmatic classification along three dimensions (i.e., functional modules, algorithmic basis, and levels of autonomy), establishing inclusion and exclusion criteria for Section 3 and revealing key considerations any rigorous trustworthiness evaluation methodology should address. A comprehensive review of automation taxonomy is beyond the scope of this paper.

### 2.1 AI‐Driven Automation in NPP (O&M): Prospects, Challenges, and Mitigation Strategies

The pursuit of AI‐driven automation in NPP O&M is driven by both economic and safety imperatives (Agarwal et al. 2022; Lin et al. 2025). In the existing nuclear reactor fleet, O&M activities account for roughly two‐thirds of total operating costs (Yadav et al. 2019), motivating automation within broader plant modernization initiatives for existing plants (Skraaning Jr et al. 2020) and advanced reactors (Holt et al. 2023). Demonstrated benefits include eliminating labor‐intensive manual processes (Al Rashdan et al. 2019; Lew et al. 2019), reducing operator cognitive burden, and improving plant operational resilience and economic viability (J.‐T. Kim et al. 2001; Naranjo de Candido et al. 2024; Sethu et al. 2023). More recently, AI‐driven systems have shown additional potential across NPP O&M contexts, including large language models (LLM) for explainable fault diagnosis (Dave et al. 2024), demonstration of ML‐based tools to anticipate equipment degradation and reduce unplanned outages (Lin et al. 2025), development of autonomous management and control systems to mitigate plant anomalies in advanced reactors (Lin, Athe, et al. 2021), explainable AI platforms to improve operator situational awareness in plant monitoring contexts (Walker et al. 2023), while other AI tools have been applied to advanced control systems supporting operator decision‐making (Cetiner et al. 2019; Hanna et al. 2020; D. Lee et al. 2018).

Successful adoption of AI‐driven automation in nuclear applications requires carefully addressing concerns raised by users, organizations, and regulators (Walker et al. 2023). User concerns include job displacement (Howard 2019), difficulty adapting to new technologies (Hoff et al. 2015), and trust miscalibration (e.g., over‐reliance or under‐reliance) on system outputs (Kohn et al. 2021; Lee et al. 2004). Organizational barriers include uncertainty in return on investment and system dependability, while regulatory challenges center on verification, validation, and licensing of autonomous systems for safety‐critical functions (Al Rashdan et al. 2021; Hall et al. 2024; Khadka et al. 2024). To mitigate these concerns, ongoing modernization programs commonly retain some form of human supervisory role, commonly described as human‐in‐the‐loop (Ali et al. 2023), human control (Tsamados et al. 2025), or human oversight (IAEA 2025; Sterz et al. 2024), keeping human operators as key decision‐makers in safety‐related contexts (Kima et al. 2018; Mortenson et al. 2024; Shneiderman 2020; Ucar et al. 2024). In addition, for plant operators, managers, and regulators, frameworks for evaluating the trustworthiness of AI‐driven technologies, justifying their safety and financial benefits are essential for overcoming institutional barriers and enabling broader adoption (Al Rashdan et al. 2021; Hall et al. 2024; Khadka et al. 2024; Wu et al. 2025). Before developing such trustworthiness evaluation frameworks, it is important to establish the scope of the AI‐driven automation systems subject to evaluation, as defined in Sub‐section 2.2.

### 2.2 Scope of AI‐Driven Automation Systems for Trustworthiness Evaluation

Trustworthiness evaluation is not system‐agnostic; the metrics, criteria, and methods used to assess trustworthiness depend fundamentally on the type of automation system under consideration and its application domain. Before reviewing existing definitions and evaluation methodologies of trustworthiness in Section 3, it is important to specify the conceptual scope of automation systems whose trustworthiness evaluation is addressed in this research. Section 3 then examines whether existing trustworthiness definitions and evaluation methods for such systems are adequate for nuclear domain.

Prior literature has proposed numerous taxonomies of automation along three conceptually distinct dimensions: functional modules, algorithmic basis, and degree of human‐automation authority allocation. With respect to functional modules (Figure 1), foundational works by Electric Power Research Institute (EPRI) (Naser 2005), aligning with Sheridan (2002), defines automation in terms of four functional components: environmental sensing, data processing and decision‐making, mechanical/informational action, and human communication. Parasuraman et al. (2000) propose a related structure with four core functions: information acquisition, analysis, decision/action selection, and implementation. Recently, the ARUGULA framework proposed by Boring et al. (2023) characterized different functions of automation according to the information‐processing stages they support, including detection, understanding, decision‐making, and action execution.

**FIGURE 1:**
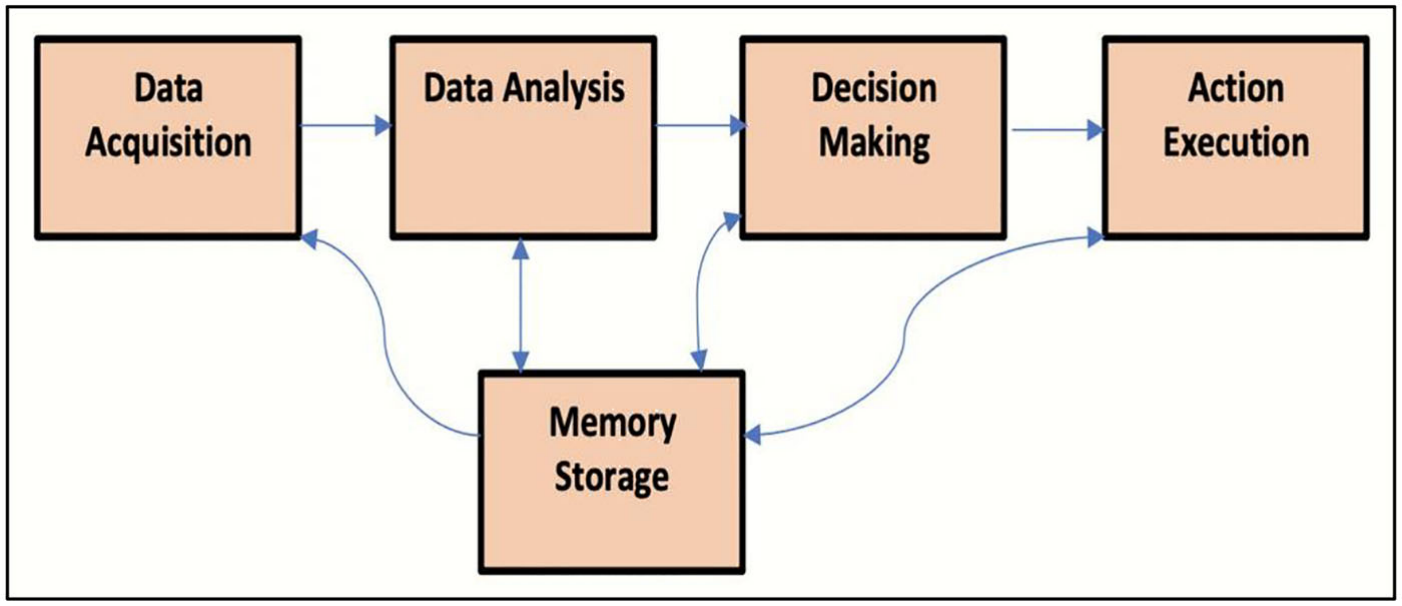
Functional modules of automation system and typical information flows among them (Boring et al. 2023; Naser 2005; Parasuraman et al. 2000; Sheridan 2002).

With respect to algorithmic basis, recent literature distinguishes rule‐based automated systems from AI‐driven autonomous systems. For example, the US NRC defined “automation” as a system that automatically takes action on a specific task according to pre‐defined, prescriptive rules, such as the actuation of reactor protection system, when process parameters exceed specified limits (NRC 2024; NRC 2023). According to Spayne et al. (2024), an automated system detects an environmental change as noted by sensors and triggers an appropriate output (e.g., an electro‐mechanical process) based on the logical sum of defined inputs. In contrast, autonomous systems determine both when and how to act based on algorithms trained on system data rather than fixed rules (NRC 2024; NRC 2023). Unlike rule‐based systems, AI‐enabled autonomous systems learn from data to recognize patterns and can make predictions that approximate aspects of human judgment (Spayne et al. 2024).

With respect to levels of automation, studies consistently propose a spectrum from fully manual to fully autonomous operation based on the allocation of responsibilities between human and system (Alberti et al. 2023; Boring et al. 2023; EPRI 2022; H. K. a. J. Kim 2025; NRC 2023; Spayne et al. 2024), though they differ in their number of levels and their characterization. For instance, (Spayne et al. 2024) allocated a separate degree to the systems manually operated from a remote location unlike (NRC 2023). Spayne et al. (2024) and NRC (2024) related autonomy to AI‐driven systems, however, *autonomous operation* in H. K. a. J. Kim (2025) and *full automation* in Alberti et al. (2023), EPRI (2022), and Boring et al. (2023) generalize these levels to any automation systems that independently perform their function without human involvement. This implies that full autonomy might be achieved with rule‐based non‐AI systems, however, such systems would not be able to perform under situations that fall outside their specified rules, unlike AI‐driven systems that can intelligently perform their intended functions based on their training. These insights suggest that while explaining taxonomy of “automation”, *degree of automation*, *functional modules*, and *algorithmic basis* are conceptually distinct and should not be conflated to avoid confusion.

This paper acknowledges that for better understanding a comprehensive taxonomy of automation is important, but it lies beyond the scope of this paper and is deferred to future work. This paper is entirely focused on trustworthiness of AI‐driven automation systems. Accordingly, this paper adopts “AI‐driven automation” as an umbrella term encompassing systems driven by AI/ML models or algorithms that employ learned, adaptive decision‐making across a spectrum of autonomy levels. The degree of autonomy exhibited by any given AI‐driven automation system depends on its functional role, its operational context, and the extent of human oversight retained in its deployment: an AI‐driven system deployed as a decision support tool exhibits autonomy limited to the recommendation layer, with human judgment governing implementation, whereas a system that both generates and executes decisions without human intervention exhibits full autonomy extending to the action execution layer.

A pragmatic classification based on the three dimensions (Table 1) defines inclusion and exclusion criteria for literature review in Section 3: a study is retained if it concerns trustworthiness of AI‐driven automation systems performing at least one functional module (Figure 1) within a broader operational process or decision‐making scenario (e.g., predicting peak cladding temperature for operator support (Lin, Bao, et al. 2021). Studies on passive data‐logging without operational influence, for example, Gallear (2019), are excluded.

**TABLE 1:** Scope of AI‐driven automation systems used in this work.

Dimension	Criterion	Example
**Types**	Algorithmic basis (AI‐driven required)	Systems driven by AI/ML algorithms or techniques in Refs. (Al Rashdan et al. 2019; Lin et al. 2025).
**Functional modules**	AI‐driven automation system must perform at least one function (Figure 1)	Data acquisition, data analysis, memory storage, decision‐making, and action execution (Boring et al. 2023; Naser 2005; Sheridan 2002).
**Levels**	Autonomy or delegation of responsibilities held by an AI‐driven automation system	Fully autonomous or Semi‐autonomous. (Alberti et al. 2023; Boring et al. 2023; EPRI 2022; H. K. a. J. Kim 2025; NRC 2023; Spayne et al. 2024)

Besides defining the inclusion criteria, the three dimensions of this classification surface three key considerations that any rigorous trustworthiness evaluation methodology should address.

- First, the functional‐modules dimension highlights that trustworthiness evaluation requires defining the system outputs under evaluation and the performance criteria they must meet.
- Second, the levels of autonomy dimension highlights that interactions among human actions, AI‐driven automation behavior, and physical phenomena (HAP) differ across autonomy levels, yet all constitute conditions under which the system must perform its intended functions. Any trustworthiness evaluation methodology must therefore capture the full HAP context rather than treating the AI‐driven automation system in isolation, to justify the appropriateness of the autonomy level under consideration.
- Third, the algorithmic basis dimension highlights that unlike rule‐based systems with largely deterministic outputs, AI‐driven systems introduce output uncertainties. These uncertainties, combined with uncertainties in human actions and physical phenomena, must be collectively characterized and quantified to measure a degree of confidence in system performance.

## 3 Existing Definitions and Evaluation Approaches for AI‐Driven Automation Trustworthiness

The literature review in this section examines how AI‐driven automation trustworthiness is defined and evaluated across domains, with emphasis on nuclear domain. The purpose is targeted: to categorize existing approaches, identify their limitations with respect to nuclear domain applicability, and establish the conceptual basis for the proposed definition and ST‐RATE methodology in Section 4. The reviewed literature concentrates in 2010–2025, when AI‐driven automation in safety‐critical domains became sufficiently mature for trustworthiness to emerge as a distinct research concern. The details on the selection of keywords and screening criteria to conduct this review are provided in Appendix A (Section A‐1) of Supporting Information. The subsections that follow organize retained studies by approach and domain per the categorization schema in Sub‐section 3.1, without restating the criteria for each study.

### 3.1 Categorization Schema of Approaches to Defining and Evaluating AI‐Driven Trustworthiness

To systematically understand the various perspectives on AI‐driven automation trustworthiness that have been discussed in the reviewed literature, this sub‐section introduces a categorization schema (Figure 2).

**FIGURE 2:**
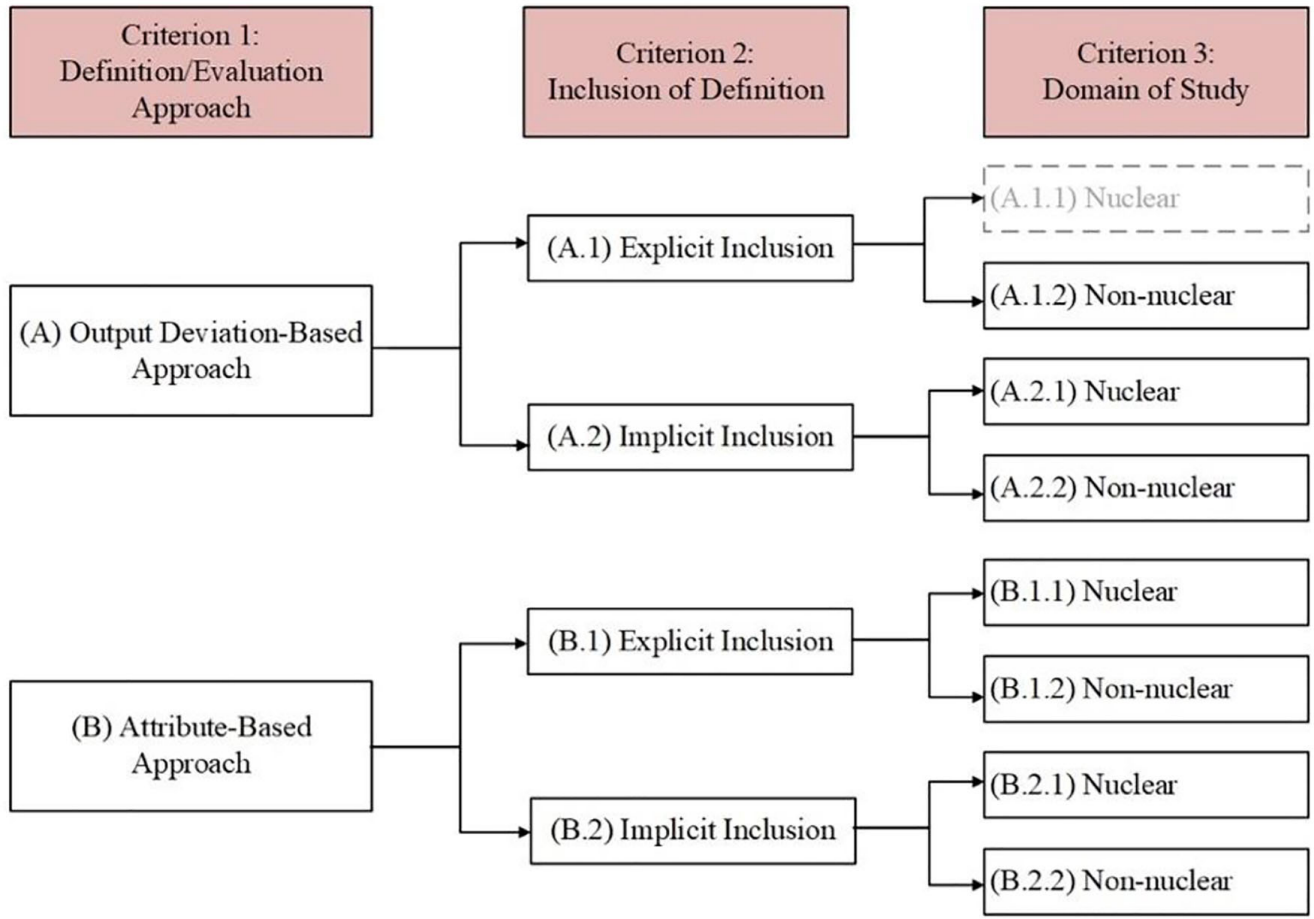
Categorization schema of AI‐driven automation trustworthiness definition and evaluation approaches. The dotted box shows that none of the reviewed studies fall under this category.

The schema classifies each reviewed study along three criteria: (1) definition/evaluation approach, (2) inclusion of definition, and (3) domain of study. The left column in Figure 2 classifies studies by definition/evaluation approach; the middle column indicates whether a definition is explicitly included or only implied in the studies; and the right column denotes the domain (nuclear vs. non‐nuclear) of the studies. Dotted block (Category A.1.1 in Figure 2) indicates that no supporting reference was found in the reviewed literature that matches the criteria for that category.

• Criterion 1: Definition/Evaluation Approach

(A) *Output deviation–based approach*: AI‐driven automation trustworthiness is articulated through observed agreement or discrepancy between AI‐driven automation outputs and reference observations (e.g., prediction–observation deviations, error/uncertainty comparisons), sometimes coupled with thresholds or acceptance bands.
(B) *Attribute‐based approach*: AI‐driven automation trustworthiness is framed as a property derived from subjective or objective attributes, such as reliability, robustness, safety, transparency/explainability, or resilience, and is often supported by qualitative justifications.

The two categories are conceptually distinct. The output deviation–based approach evaluates trustworthiness directly from measurable discrepancies between system outputs and reference observations, requiring neither predefined attributes nor aggregation, as the deviation metric itself serves as the trustworthiness measure. In contrast, the attribute‐based approach treats trustworthiness as an overarching property derived from selected system characteristics that are individually assessed and aggregated into an overall evaluation, without necessarily requiring observed output data.

• Criterion 2: Inclusion of Definition

(1) *Explicit inclusion*: The study states a definition of AI‐driven automation trustworthiness (original or cited) and uses it to motivate methods, indicators, or thresholds. Such explicit definitions were analyzed to inform the proposed definition and concepts in Section 4.
(2) *Implicit inclusion*: The study uses the concept of AI‐driven automation trustworthiness without stating a definition, typically via an evaluation method (e.g., error‐based comparison, attribute checklist) that implies what “trustworthiness” entails.

• Criterion 3: Domain of Study

(1) *Nuclear*: Studies in nuclear contexts (e.g., NPP operations and maintenance) that address AI‐driven automation trustworthiness.
(2) *Non‐nuclear*: Cross‐domain studies (e.g., robotics, automotive, energy systems, and other safety‐critical domains) that offer transferable insights on AI‐driven automation trustworthiness.

This schema provides a structured overview of trustworthiness definitions and evaluation approaches in the literature. It is used to categorize the references reviewed in Sub‐sections 3.2 and 3.3 and to identify gaps in Sub‐section 3.4 that motivate the definition proposed in Section 4.

### 3.2 Output Deviation–Based Approach

This sub‐section reports key findings from studies reviewed under Category A (Figure 2) that adopted an output deviation–based approach to define or evaluate AI‐driven automation trustworthiness. Studies are grouped by explicit versus implicit inclusion of a definition and by domain (nuclear vs. non‐nuclear), consistent with Figure 2. Section A‐2 (see Appendix A of Supporting Information) synthesizes the key findings from these studies.

No reviewed studies were found in Category A.1.1 (explicit, nuclear), underscoring a gap in the literature where explicit, output deviation–based definitions of trustworthiness have yet to be formalized for nuclear applications. In Category A.1.2 (explicit, non‐nuclear), studies such as Taddeo (2010) and Sun et al. (2016) formalize trustworthiness through measurable deviations, offering clear, mathematically grounded definitions and metrics; however, their reliance on extensive empirical data, user‐defined thresholds, and lack of integration with regulatory practices limit transferability to nuclear contexts. In Category A.2.1 (implicit, nuclear), studies by Lin, Bao, et al. (2021), Hanna et al. (2021), and Y.‐J. Kim et al. (2021) demonstrate the practical use of discrepancy checkers, reconstruction errors, and reasoning‐based metrics to operationalize trustworthiness in nuclear settings ranging from digital twins to operator support systems and fire detection. These works provide valuable nuclear‐domain demonstrations, including fail‐safe triggers, real‐time reasoning, and use of validation test data in NRC‐relevant experiments, but are limited by empirically set thresholds, dependence on training‐domain coverage or synthetic data, and the absence of explicit linkage to plant‐level safety risk acceptance criteria. Finally, Category A.2.2 (implicit, non‐nuclear) studies such as Chen et al. (2021) and Rahmati et al. (2023) extend the output deviation–based approach to PLC integrity and nano‐lubricant modeling, respectively, showing strong empirical validation and adversarial robustness, yet sharing common limitations of being confined to the scope of available data, empirically set thresholds, and lack of uncertainty quantification.

Overall, Category A studies illustrate the strengths of the output deviation–based approach in offering transparent, quantitative, and easily interpretable mechanisms for trustworthiness evaluation, but also reveal consistent limitations with respect to their adoption in the nuclear domain that are synthesized in Section A‐2 (See Appendix A of Supporting Information) and summarized in Sub‐section 3.4.

### 3.3 Attribute‐Based Approach

This sub‐section reports key findings from studies reviewed under Category B (Figure 2) that adopted an attribute‐based approach to define or evaluate AI‐driven automation trustworthiness, grouped by explicit versus implicit inclusion of a definition and by domain, consistent with Figure 2. Section A‐3 (see Appendix A of Supporting Information) synthesizes the key findings from these studies.

In Category B.1.1 (explicit, nuclear), a strong consensus was observed on “reliability” or “correctness” as the most critical attributes of trustworthiness, followed by safety and resilience, underscoring the expectation that AI‐driven systems should operate reliably, maintain overall safety, and perform robustly under varying operational conditions. The attribute of explainability was absent in earlier works (e.g., Smidts et al. [2019]; (Muhlheim et al. 2022) but appeared consistently in recent studies (e.g., Wang [2022], Saboury et al. [2023], and Wu et al. [2025]), highlighting its growing importance in recent research. In Category B.1.2 (explicit, non‐nuclear), Sheridan (2019) provided one of the earliest systematic formulations of trustworthiness, later distinguishing trustworthiness, an objective property of automation systems, from trust, a subjective property of users (Sheridan 2019). He proposed reliability, robustness, and understandability as objectively measurable attributes of trustworthiness, and familiarity and dependence as subjective attributes of user trust. Across this category, reliability and safety remained the most common attributes, while explainability gained consistency in studies over the past six years, similar to Category B.1.1. However, privacy and fairness also emerged as prominent attributes in several non‐nuclear domains, which were found to be absent in the studies under Category B.1.1.

Across Category B.2.1 (implicit, nuclear), reliability consistently emerged as a fundamental attribute of trustworthiness. Elks et al. (2019) and Kobayashi and Alam (2024) emphasized that trustworthy AI‐driven systems must be resilient and capable of meeting user expectations under challenging conditions. While Agarwal et al. (2022) and Alberti et al. (2023) highlighted explainability as an essential attribute, Kobayashi and Alam (2024) uniquely conceptualized trustworthiness itself as a characteristic of explainable AI. Agarwal et al. (2022) also identified efficiency as a key factor, whereas Mortenson et al. (2024) underscored the need for independent oversight (e.g., from the IAEA) to ensure trustworthiness. Walker et al. (2023) notably introduced the “trust but verify” concept, encouraging users to critically evaluate system decisions to sustain informed trust in plant monitoring. Finally, Kollias et al. (2022) highlighted the importance of uncertainty quantification and confidence estimation in machine learning predictions as a means to foster trustworthy AI.

Finally, studies in Category B.2.2, frequently identified resilience as a fundamental basis to system trustworthiness, with Sifakis (2019) linking it to an autonomous system's ability to withstand diverse hazards, and Mandal et al. (2021) relating it to a model's capacity to generalize to unseen data. Studies such as Shneiderman (2020), Hall et al. (2024), and Henderson et al. (2022) further associated system trustworthiness with compliance to standards and oversight by bodies like ISO, IEEE, FDA, and FAA. Attributes such as explainability, interpretability, and transparency were also interchangeably used for system trustworthiness (e.g., Karra et al. [2021], Mahdavifar [2021], Buluc et al. [2021], Volkova et al. [2022]). Reliability and safety were consistently cited as core pillars of trustworthy systems (e.g., Shore et al. [2021], Fisher et al. [2021], Findrik et al. [2017]), while reproducibility and repeatability were also highlighted as indicators of trustworthiness (Ahmed et al. 2021). Broader theoretical frameworks, such as those proposed by Ersöz et al. (2022) and Zafer et al. (2024), integrated both social (justice, accountability, value congruence) and technical (robustness, repeatability, explainability) dimensions, underscoring that trustworthy AI must operate ethically, transparently, and safely across its entire lifecycle.

Overall, several thematic observations emerge across the reviewed studies as illustrated in Figure 3. Explainability is the most consistently cited attribute across both nuclear and non‐nuclear studies, followed by Reliability and Safety. Explainability remained absent in earlier works (Muhlheim et al. 2022; Smidts et al. 2019) but appears consistently in recent studies (Saboury et al. 2023; Wang 2022; Wu et al. 2025). Attributes such as Security, Privacy, Robustness, Fairness, and Accountability appear prominently in non‐nuclear domains but are largely absent from nuclear‐focused studies, reflecting domain‐specific differences in what trustworthiness requires. Generalizability, Dependability, Agility, and Performability were the least common attributes across reviewed studies, appearing exclusively in non‐nuclear domains. Explainability, interpretability, and transparency are frequently conflated in non‐nuclear studies without clear distinction.

**FIGURE 3:**
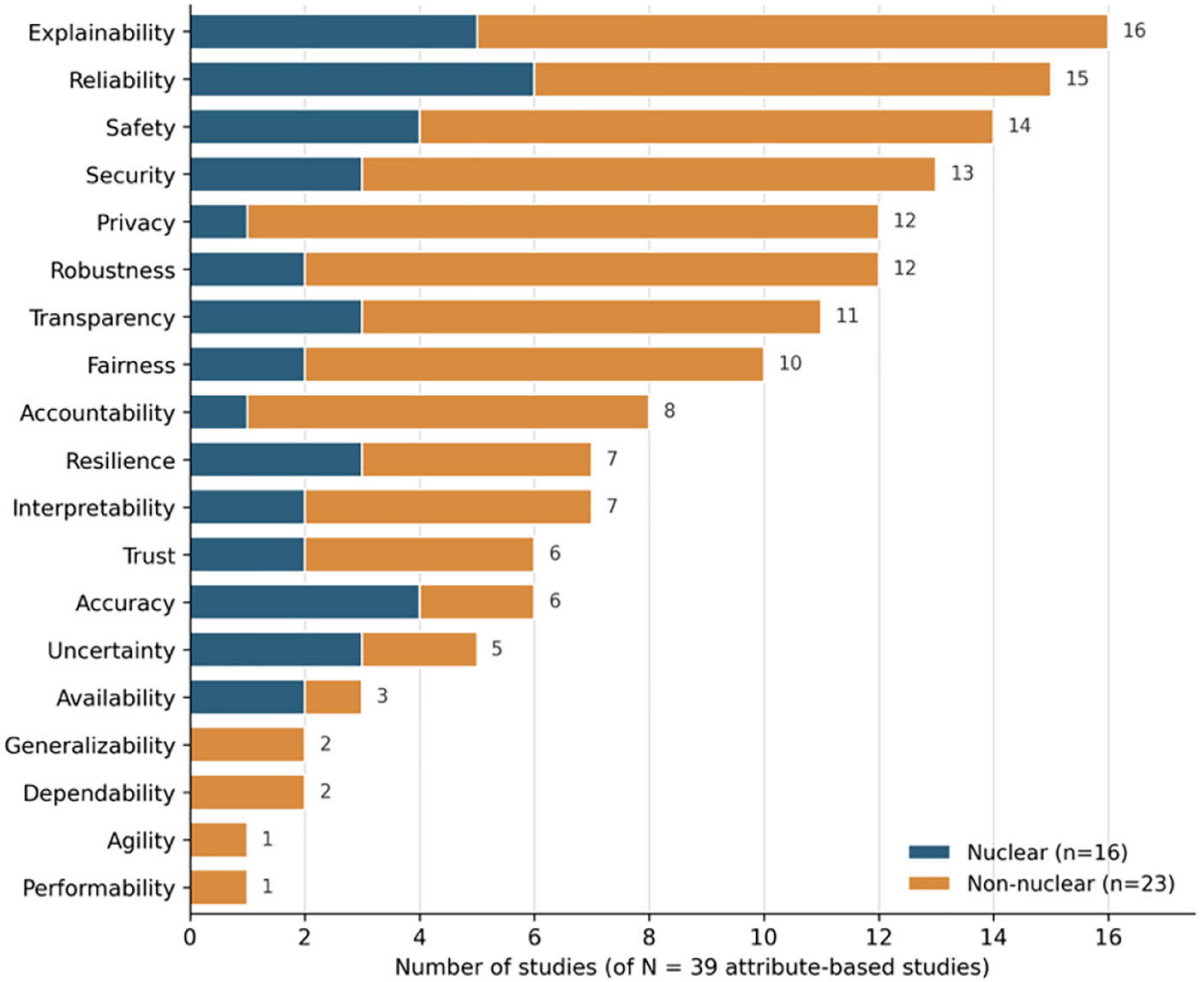
Frequency of trustworthiness attributes in the reviewed studies (Section A‐3 in Appendix A of Supporting Information).

Several studies under the attribute‐based approach (Category B, Figure 2) have operationalized their definitions of trustworthiness by proposing methods or frameworks to measure, assess, or enhance the trustworthiness of AI‐driven systems. These efforts can be broadly classified into three groups.

• First, some studies proposed methods to measure specific attributes of trustworthiness rather than the overall system trustworthiness. For instance, Smidts et al. (2019) introduced mathematical models to quantify the reliability and availability of industrial control systems (ICSs) but did not leverage these measurable attributes to evaluate overall trustworthiness of ICS.
• Second, several studies embedded trustworthiness attributes directly into the design and implementation of AI‐driven systems. Zhou et al. (2023) employed the Dempster–Shafer evidence theory to quantify belief mass and uncertainty in deep neural networks (DNNs), enhancing robustness in out‐of‐distribution scenarios. Franco (2024) integrated privacy and fairness into model algorithm using techniques such as homomorphic encryption and fair data representation. Roszel (2024) proposed the Know Your Model (KYM) framework, outlining lifecycle‐wide qualitative guidelines to promote transparency, accountability, and confidence in AI systems. Similarly, Manchanda (2022) introduced an End‐to‐End Agile and Automated ML Framework that embeds quality‐assurance checkpoints, bias detection, and continuous monitoring to enhance model trustworthiness throughout its lifecycle. Kobayashi and Alam (2024) utilized quantitative metrics (e.g., MSE, split conformal prediction, covariate perturbation) to evaluate accuracy, robustness, and resilience in different models to compare their trustworthiness under similar conditions. Lin et al. (2025) enhanced explainability through post‐hoc interpretation methods such as SHAP and LIME, while Ahmed et al. (2021) demonstrated repeatability by controlling random parameter generation during model training. By embedding various attributes into the algorithms of AI‐driven systems, these studies characterized their models as trustworthy without developing a quantitative metric or a structured framework to explain the relative contribution of each attribute to overall system trustworthiness. However, to determine whether the achieved level of automation trustworthiness meets the acceptable thresholds defined by independent regulatory bodies, the development of a quantitative trustworthiness metric is essential.
• Third, very limited number of studies attempted to establish quantitative metrics of trustworthiness under the attribute‐based approach. Sheridan (1989) proposed that reliability, usefulness, and robustness could be measured and combined multiplicatively to represent overall trustworthiness, though this lacked formal validation. More rigorously, Wang (2022) developed a Bayesian Belief Network (BBN)‐based framework to quantify the trustworthiness of the NAMAC system during its design and operation phases. In this work, theoretical causal models were first developed to capture both direct and indirect relationships among various trustworthiness attributes and the overall system trustworthiness. These causal models were then transformed into bottom‐up Bayesian Belief Network (BBN) models, with trustworthiness represented as the top node. The BBN models were subsequently used to quantify trustworthiness through Conditional Probability Tables (CPTs) derived via expert elicitation. Within this framework, NAMAC's development‐phase trustworthiness was measured to be 78%. Wang (2022) proposed a well‐structured framework to evaluate a quantitative metric of trustworthiness within the attribute‐based approach. However, to enable a more accurate and realistic estimation of trustworthiness, trustworthiness evaluation should also consider addressing potential biases arising from expert judgment and inclusion of uncertainty analysis related to the operational and environmental conditions under which the automation system may be deployed, thereby broadening its applicability across dynamic contexts and safety‐critical domains. Furthermore, comparing the degree of trustworthiness for a given context against predefined acceptance thresholds is as crucial as measuring it, particularly for justifying the deployment feasibility of automation systems in highly regulated and safety‐critical domains such as nuclear power.

Similar to output deviation–based approach, studies using the attribute‐based approach also share limitations with respect to their applicability in the nuclear domain that are synthesized in Section A‐3 (See Appendix A of Supporting Information) and summarized in Sub‐section 3.4.

### 3.4 Critical Gaps in Existing Approaches and the Conceptual Basis for ST‐RATE

Taken together, neither the output deviation–based nor the attribute‐based approach alone is sufficient for trustworthiness evaluation in the nuclear domain.

Studies in Sub‐section 3.2 illustrate the quantifiability of trustworthiness under the output deviation–based approach but consistently reveal four nuclear‐specific gaps (Section A‐2 in Appendix A of Supporting Information): (1) dependence on large, domain‐specific empirical datasets that are often scarce or proprietary in NPP settings; (2) thresholds lacking regulatory anchoring with no connection to plant‐level risk acceptance criteria such as core damage frequency; (3) insufficient consideration of socio‐technical operating context in performance evaluation; and (4) insufficient integration with uncertainty quantification and risk‐informed safety criteria.

Studies in Sub‐section 3.3 demonstrate the conceptual breadth of attribute‐based approach in characterizing trustworthiness across diverse system types and lifecycle stages, but consistently reveal four nuclear‐specific gaps (Section A‐3 in Appendix A of Supporting Information): (1) no consensus on a set of trustworthiness attributes for AI‐driven systems, even for a specific application, and no guidance on how relevant attributes may vary across system types, functional roles, and application contexts justifying their comprehensiveness to characterize trustworthiness; (2) subjectivity or absence of quantitative methods for measuring individual attributes; (3) no quantitative model linking attributes to an overall trustworthiness metric; and (4) no mechanism for comparing achieved trustworthiness against regulatory acceptance thresholds to justify nuclear deployment.

These gaps collectively motivate the conceptualization of a new definition and ST‐RATE methodology in Section 4, grounded in three pillars: *Pillar 1* addresses attribute appropriateness and context‐dependence by shifting from a fixed attribute set to application‐specific, quantifiable performance metrics, spanning safety, operational, regulatory, and economic dimensions, each compared against its corresponding acceptance threshold; *Pillar 2* explicitly models the socio‐technical operating context omitted from all reviewed studies; and *Pillar 3* addresses data scarcity and subjectivity concerns through formal uncertainty‐based quantification of trustworthiness comparable against domain‐specific acceptance thresholds.

## 4 Proposed Definition for AI‐Driven Automation Trustworthiness

The literature review in Section 3 illustrates both the diversity and commonalities in how AI‐driven automation trustworthiness is defined and measured. It also reveals gaps across both output deviation–based and attribute‐based approaches with respect to their adoption in the nuclear domain. Despite the growing integration of AI‐driven automation in nuclear applications (Cetiner et al. 2019; Dave et al. 2024; Hanna et al. 2020; D. Lee et al. 2018; Lin, Athe, et al. 2021; Lin et al. 2025; Walker et al. 2023), no unified definition of trustworthiness exists that adequately addresses the nuclear domain's specific challenges (Sub‐section 3.4).

Building on the key considerations established through the pragmatic classification in Sub‐section 2.2 and the gaps identified in Sub‐section 3.4, this section proposes a new definition of AI‐driven automation trustworthiness and conceptualizes the ST‐RATE methodology tailored for the nuclear domain. The definition proposed in this work does not fully reflect the output deviation–based or attribute‐based approaches; rather, it is designed to: (a) be applicable for a wide range of automation technologies, applications and contexts within the nuclear domain; (b) address the inherent challenges in existing definitions of AI‐driven automation trustworthiness that are associated with empirical data scarcity, subjectivity and justifying the appropriateness of attribute sets, absence of regulatory anchoring, and insufficient consideration of socio‐technical operating context; and (c) develop a conceptual basis facilitating the development of a risk‐informed, systematic, and scientifically justifiable methodology for AI‐driven automation trustworthiness evaluation. This research proposes a new definition of AI‐driven automation trustworthiness as:

***“The*** *degree of confidence* ***that the AI‐driven automation system will function*** *as expected across its conditions of use* ***.”***

The definition and the ST‐RATE methodology are grounded in three foundational pillars, each corresponding to a key phrase in the definition and addressing key considerations and gaps identified in Sub‐sections 2.2 and 3.4.

• **Pillar #1 “As Expected”—Risk‐Informed Performance Expectations**:

The phrase “as expected” in the proposed definition implies the existence of predefined performance expectations and associated acceptance thresholds. These performance expectations and their thresholds may pertain to safety metrics (e.g., core damage frequency in safety risk assessment of NPPs), operational criteria (e.g., false alarm rates, detection times), regulatory criteria (e.g., maximum annual fire‐induced core damage frequency), or economic metrics (e.g., cost or benefit thresholds). Importantly, this expectation is not merely about functional requirements; rather, it also encompasses the avoidance of undesirable consequences especially those involving safety and financial impacts. Performance expectations and acceptance thresholds should be defined in close collaboration with key stakeholders involved in the design, development, and deployment of AI‐driven automation systems, including operators, plant managers, and regulators, to ensure their alignment with operational realities, regulatory requirements, and organizational priorities. This process strengthens the technical rigor of the trustworthiness evaluation while enhancing stakeholder trust in AI‐driven systems, supporting their adoption.

For example, in the case of an AI‐AFW system being deployed for a safety‐critical application in NPPs, this expectation includes not only the ability to detect fires accurately within predefined performance criteria, but also the ability to avoid missed detections (e.g., those arising from rare events outside the training domain of the AI‐AFW system) which could lead to undetected fire growth or false alarms that may result in unnecessary evacuations and/or operational disruptions. In such context, trustworthiness involves ensuring that the AI‐driven automation system contributes to maintaining plant safety and operational continuity and does not introduce new or unacceptable risks through failure modes or performance degradations, especially those not anticipated during design and validation. The types of risk and the acceptable levels of risk depend on the application context in which the AI‐driven automation system is used. Therefore, the term “as expected” establishes a direct linkage between the performance of the AI‐driven automation system and the domain‐ or application‐specific acceptability criteria that govern the decision to trust and deploy the system. Pillar #1 of the proposed definition of AI‐driven automation trustworthiness is related to the “risk‐informed” element of the ST‐RATE methodology (Khalid et al. 2026). The risk‐informed perspective supports defensible decision‐making, where acceptance or rejection of AI‐driven automation technologies is based on quantified contributions to system‐level risk and compliance with established acceptance criteria. This approach provides a structured path for decision‐makers (e.g., operators, plant managers, regulators) to reason about the deployment of AI‐driven automation.

Pillar 1 is operationalized in Khalid et al. (2026) through an Integrated‐Probabilistic Risk Assessment (I‐PRA) architecture that connects automation trustworthiness evaluation directly with plant PRA. Performance acceptance thresholds are defined in terms of quantifiable plant safety risk metrics (e.g., fire‐induced core damage frequency), anchoring the trustworthiness evaluation to risk‐informed insights that directly guide AI‐driven automation system design, development, and deployment decision making. Pillar 1 addresses the consideration surfaced by the functional modules dimension (Sub‐section 2.2), by carefully defining the outputs of an AI‐driven automation system that are subject to evaluation and the performance criteria they are expected to satisfy for trustworthiness evaluation. Importantly, these performance expectations and associated acceptance thresholds should not be restricted to a predefined set of trustworthiness attributes for a range of applications, as highlighted in Sub‐section 3.4; rather, they should be application‐specific and encompass quantifiable safety, operational, regulatory, and economic metrics relevant to the intended use of the AI‐driven automation system.

• **Pillar #2 “Across Its Conditions of Use”—Socio‐Technical Context**:

The term “across its conditions of use” in the proposed definition refers to the range of operational and environmental conditions in which the AI‐driven automation is deployed. For example, in the AI‐AFW, this may include physical environment factors (such as lighting and ambient temperature), variations in human (such as operators or first responders) behavior and decision‐making, as well as the dynamic evolution of physical phenomena such as fires or equipment degradation. These are not isolated factors but are interdependent and form the socio‐technical context in which AI‐driven automation systems must operate. Pillar 2 grounds the socio‐technical element of the ST‐RATE methodology (Khalid et al. 2026). The ST‐RATE methodology explicitly models Human‐Automation‐Physical (HAP) phenomena to capture these socio‐technical factors and their interactions with the AI‐driven automation system, allowing for evaluating how they influence system performance.

Pillar 2 ensures that trustworthiness is evaluated across the full spectrum of scenarios, the AI‐driven system may encounter during deployment across its conditions of use. This includes not only expected operating conditions but also low‐probability, high‐consequence scenarios that often provide the most rigorous test of system performance and robustness. These conditions of use are characterized through probability distributions associated with the inputs to the HAP coupled model, collectively representing the range of plausible scenarios that may arise during system operation. Each sampled set of HAP model inputs from these distributions represents a scenario. During the training phase, the AI model is trained using a representative subset of scenarios drawn from these distributions, primarily reflecting nominal operating conditions. During the application phase, system performance is evaluated over a substantially larger set of samples drawn from the same distributions, encompassing both nominal conditions represented within the training domain and off‐nominal conditions not encountered during training, including rare but plausible scenarios sampled from these distributions.

Pillar 2 is operationalized in Khalid et al. (2026) through a unified simulation platform that couples human, AI‐driven automation, and physical phenomena models (HAP) and integrate them with plant PRA model. This integration allows for the propagation of socio‐technical uncertainties from the underlying HAP coupling up to the level of plant safety risk metrics. Pillar 2 addresses the consideration surfaced by the levels of autonomy dimension (Section 2.2), which recognizes that the HAP interactions differ across autonomy levels, yet all constitute conditions under which the system must satisfy the performance expectations defined under Pillar 1. The HAP integrated simulation platform developed under Pillar 2, therefore capture the full HAP context rather than treating the AI‐driven automation system in isolation. Importantly, this relationship is bidirectional: the autonomy level shapes the socio‐technical context that trustworthiness evaluation must model, while the resulting trustworthiness assessment should in turn inform what autonomy level is operationally defensible for a given application.

• **Pillar #3 “Degree of Confidence”—Uncertainty‐Based Trustworthiness Evaluation**:

The term “degree of confidence” in the proposed definition embeds the concept of uncertainty and forms the quantitative and probabilistic basis for evaluating AI‐driven automation trustworthiness. In this definition, the degree of confidence is not a subjective impression, but a measurable indicator obtained through uncertainty analysis. Specifically, the degree of confidence that the AI‐driven automation system will perform as expected under its intended conditions of use is assessed by quantifying the uncertainty associated with the probability that its performance meets defined acceptance thresholds. This assessment accounts for both epistemic and aleatory uncertainties related to operating conditions, performance mechanisms, and the thresholds themselves. As mentioned under Pillar #1, these thresholds are determined based on specific objectives and requirements of the intended application; for instance, ensuring that fire‐induced core damage frequency remains below regulatory limits in an NPP Fire safety risk scenario.

In the ST‐RATE methodology, to operationalize Pillar# 3, the uncertainty analysis is conducted to quantify the total uncertainty (i.e., including both aleatory and epistemic sources of uncertainty) in the performance output of the AI‐driven automation system (Khalid et al. 2026). The uncertainty analysis must account for the full spectrum of socio‐technical factors (captured by the HAP simulation in ST‐RATE), which reflect variations in human actions, AI‐driven automation responses, and physical phenomena within the defined conditions of use. The resulting total uncertainty distribution is then compared against the application‐specific acceptance thresholds to quantify (i) the probability that the system performance satisfies the defined acceptance thresholds, and (ii) the uncertainties associated with this probability. The results help determine whether the AI‐driven automation system is sufficiently trustworthy for its intended application. This assessment should be conducted offline at the design time to support deployment decision‐making, considering the following guidelines (Figure 4).

- If the total uncertainty in the performance output remains within the acceptable thresholds (Case 1 in Figure 4), the AI‐driven automation model is considered fully trustworthy (with 100% confidence) for that specific application, under the given conditions and predefined acceptable risk or threshold levels
- If the total uncertainty lies entirely outside the acceptable thresholds (Case 2 in Figure 4), the model is deemed untrustworthy with 100% confidence for that specific application, under the given conditions and predefined acceptable risk or threshold levels. In such cases, one may choose to modify the design to improve both the AI‐driven automation's trustworthiness and the overall system performance.
- When the total uncertainty overlaps with acceptable threshold (Case 3 in Figure 4), the probability of not exceeding that threshold, and its associated epistemic uncertainty, represents the level of trustworthiness in the AI‐driven automation for that specific application, under the given conditions and predefined acceptable risk or threshold levels. If this probability is not large enough to support deployment decision‐making, further steps may be taken to reduce epistemic uncertainty (e.g., through additional data collection or by increasing the resolution of performance models) to determine more clearly whether the total uncertainty lies within the acceptable threshold. Alternatively, the system design may be adjusted to ensure the system's performance meets the threshold requirements.

**FIGURE 4:**
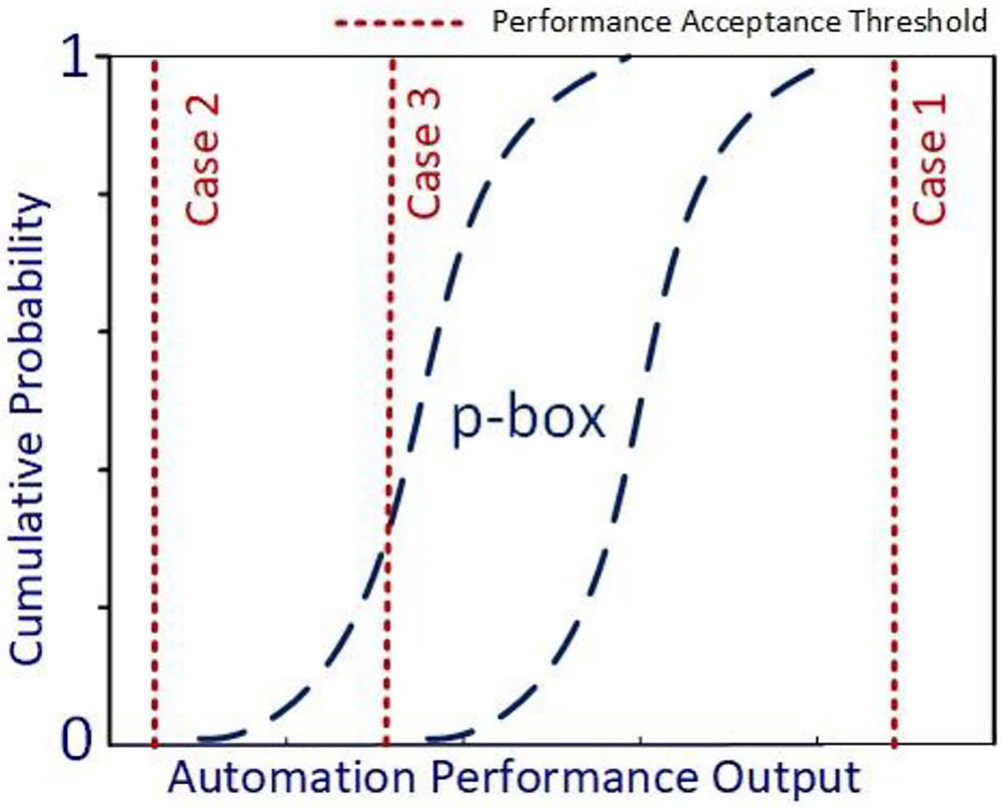
AI‐driven automation trustworthiness evaluation scenarios.

Pillar 3 addresses the consideration identified by the algorithmic‐basis dimension (Sub‐section 2.2): unlike rule‐based systems with largely deterministic outputs, AI‐driven systems rely on learned models that introduce output uncertainties. These uncertainties, together with uncertainties in human actions and physical phenomena, are propagated through the HAP‐coupled simulation to quantify a probabilistic measure of confidence that the system satisfies its acceptance criteria across its conditions of use.

While existing studies emphasize the importance of VVUQ in developing trustworthy AI‐driven automation systems (Bradshaw et al. 2022; Kollias et al. 2022; Salunkhe 2023; Wu et al. 2025; Zhou et al. 2023), they fall short of articulating a clear methodological bridge between VVUQ practices and trustworthiness evaluation, particularly due to subjectivity in certain attributes such as interpretability and explainability. By combining risk‐informed acceptance thresholds, socio‐technical modeling, and formal uncertainty analysis, ST‐RATE addresses the limitations of both output deviation–based and attribute‐based approaches identified in Sub‐section 3.4, remaining effective even with limited empirical data and minimizing reliance on subjective expert judgment.

## 5 Concluding Remarks

This paper presents a cross‐disciplinary review of existing definitions and methodologies for AI‐driven automation trustworthiness, analyzes their strengths and limitations for applications in nuclear domain, proposes a new definition of AI‐driven automation trustworthiness to address identified limitations, and conceptualizes a novel risk‐informed trustworthiness evaluation methodology, namely the ST‐RATE.

The cross‐disciplinary review identified two main approaches to defining and measuring AI‐driven automation trustworthiness: output deviation–based and attribute‐based. The output deviation–based approach measures trustworthiness from bounded discrepancies between AI‐driven automation outputs and reference observations. Although this approach aligns well with empirical validation logic, its reliance on large, scenario‐specific datasets limits its applicability in the nuclear domain, particularly in safety‐critical applications where data are scarce or proprietary. The attribute‐based approach defines trustworthiness through a set of system attributes such as reliability, explainability, and robustness. While useful when observational data are limited, this approach faces challenges in defining a scientifically justifiable attribute set to characterize trustworthiness and in addressing subjectivity in measuring individual attributes. Furthermore, neither approach provides a mechanism for comparing achieved trustworthiness against regulatory acceptance criteria, a requirement essential for nuclear deployment decisions. It is therefore concluded that neither approach alone is adequate, and a more practical, quantitative definition of AI‐driven automation trustworthiness is needed.

This paper fills this gap and proposes a new definition of AI‐driven automation trustworthiness as “the degree of confidence that the AI‐driven automation system will function as expected across its conditions of use.” Based on this definition, a novel methodology, namely the ST‐RATE, is conceptualized. The proposed definition and the ST‐RATE method are grounded in three key pillars. Pillar 1 is related to the term “as expected” in the proposed definition and highlights the existence of predefined performance expectations and their associated acceptance thresholds. Pillar 1 aligns with the risk‐informed element of the ST‐RATE method. Pillar 2 is related to the term “across its conditions of use” in the proposed definition and refers to the range of operational and environmental conditions under which the AI‐driven automation is deployed. Pillar 2 aligns with the socio‐technical element of the ST‐RATE methodology. Pillar 3 is related to the term “degree of confidence” in the proposed definition that offers a measurable indicator to evaluate AI‐driven automation trustworthiness obtained through uncertainty analysis. Pillar 3 establishes an uncertainty‐based foundation for the ST‐RATE methodology to address the key limitations of existing approaches by enabling effective quantification of trustworthiness despite limited empirical data and reducing reliance on subjective expert judgment.

Pillars 1 and 2 are intentionally extensible. Performance thresholds under Pillar 1 are application‐specific and can be expanded, in collaboration with relevant stakeholders, to include additional metrics (e.g., security‐related criteria) as needed. Conditions of use under Pillar 2 can similarly be updated as deployment knowledge grows or previously unconsidered conditions, including adversarial ones, are identified. ST‐RATE thus does not claim to offer a complete or universal account of AI trustworthiness; rather, it provides a structured, risk‐informed, quantitative framework designed to evolve with stakeholder requirements and deployment context. While ST‐RATE framework is developed primarily for the nuclear domain, the proposed definition and ST‐RATE methodology has the potential to be adapted to other safety‐critical domains with appropriate modifications. The development of ST‐RATE framework, and its demonstration through an AI‐AFW case study, are presented in Khalid et al. (2026).

## Conflicts of Interest

The authors declare no conflicts of interest.

## Supporting information




**Supporting Information**: risa70367‐supp‐0001‐SuppMat.docx

## Data Availability

Data such as relevant references identified and/or analyzed during the entire literature review process are available from the corresponding author on reasonable request.
