# Supplementary material for: Socio‐Technical Risk‐Informed AI‐Driven Automation Trustworthiness Evaluation (ST‐RATE): Part 1‐Conceptualization
Source: Risk Anal. 2026 Oct 7;46(11):e70367. doi: 10.1111/risa.70367 (PMC13643605; doi:10.1111/risa.70367)
Supplement: Supplementary file 1 [file RISA-46-0-s001.docx]

**Socio-Technical Risk-informed Automation Trustworthiness Evaluation (ST-RATE): Part 1-Conceptualization - Appendix A**

The appendix A covers the following sections. Please refer to the main manuscript for relevant details.

1. **Section A-1**: This section explains the selection of keywords and screening criteria that drives the literature review on the definitions and methodologies of AI-driven Automation Trustworthiness
2. **Section A-2**: This section provides the synthesis of existing definitions and evaluation methodologies for AI-Driven Automation Trustworthiness using Output-Deviation-based approach, corresponding to Sub-section 3.2 in the main-manuscript.
3. **Section A-3**: This section provides the synthesis of existing definitions and methodologies of AI-Automation Trustworthiness using the Attribute-based approach, corresponding to Sub-section 3.3 in the main-manuscript.

- *Section A-1: Selection of Keywords and Screening Criteria to Conduct the Literature Review*

Initial exploratory searches using broad keyword combinations yielded a large volume of results, many only tangentially related, often referencing nuclear topics without substantive focus on trustworthiness of AI-driven systems. To increase conceptual centrality and domain relevance, a top-down keyword refinement approach (Table A-1) was adopted, returning approximately 2,000 records screened against the following inclusion and exclusion criteria, consistent with Sub-section 2.2 in the main manuscript:

Table A-1: Top-down Keyword Refinement Approach for Literature Search

| **Search Strategy** | **Search Terms / Keywords** | **Purpose** |
| --- | --- | --- |
| 1. *Title-restricted, nuclear queries* | nuclear AND (artificial intelligence OR automation OR machine learning) | Prioritize domain relevance (nuclear context central) |
|  | nuclear AND (artificial intelligence OR automation OR machine learning) AND (trust OR trustworthy OR trustworthiness) | Prioritize conceptual centrality of trustworthiness in AI-driven nuclear studies |
|  | nuclear AND (artificial intelligence OR automation OR machine learning) AND (review OR literature review) | Identify nuclear syntheses and use these syntheses to refine/confirm definitions/frameworks |
| 2. *Expanded nuclear queries* | Title: nuclear.  Full text: (artificial intelligence OR automation OR machine learning) AND (trust OR trustworthy OR trustworthiness) | Recover nuclear papers where trustworthiness is discussed contextually (not necessarily in title) |
| 3. *Cross-domain, title-only queries* | (artificial intelligence OR automation OR machine learning) AND (trust OR trustworthy OR trustworthiness) AND (automobile OR medicine OR robotics OR energy generation) | Provide comparative perspective from other domains (e.g., medicine, automotive, energy generation) to inform the schema; excluded from synthesis unless about AI-driven. |
| 4. *Backward citation tracing* | References from retrieved papers | Reduce missed relevant references. |

- **Inclusion criteria:** A reference is retained if it satisfies: (i) AI/ML-based automation; (ii) connection to at least one functional module in Table 1 and Fig. 1; and (iii) relevance to an operational process or decision context (e.g., O&M, operator decision support).
- **Exclusion criteria:** Studies concerning non-AI automation (e.g., rule or control theory based) are excluded. Additionally, studies within domains such as IoT and cybersecurity that frame trustworthiness as a relational property between entities (i.e., conflating "trust" and "trustworthiness") are excluded (e.g., (Ma et al., 2021; Ugur, 2021); among others). In the present work, trustworthiness is treated as an intrinsic property of the AI-driven automation system (the trustee), distinct from trust, which is a property of the user (the trustor), consistent with (Sheridan, 2019a). Studies focused on human perception of trust rather than the system property of trustworthiness are therefore excluded. Some relevant studies may have been inadvertently excluded due to alternative terminology conceptually related to trustworthiness but absent from the search keywords (e.g., 'trustability' (Hanna et al., 2021; Kim et al., 2025))

It should be noted that the purpose of this literature review is targeted: to categorize existing approaches, identify their limitations with respect to nuclear domain applicability, and establish the conceptual basis for the proposed definition and ST-RATE methodology in Section 4. It is not intended as an exhaustive review of all AI-driven automation articles nor as a quantitative meta-analysis of AI-driven automation trustworthiness literature.

- *Section A-2: Synthesis of Existing Definitions and Evaluation Methodologies for AI-Driven Automation Trustworthiness Using Output-Deviation-Based Approach*

| **Study** | **Context** | **Key Insights** | **Limitations for Nuclear Deployment** |
| --- | --- | --- | --- |
| ***(A.1.2) Output Deviation-based – Explicit Definition – Non-Nuclear Domain*** | | | |
| (Taddeo, 2010); later acknowledged by (Schmid et al., 2022) | Distributed digital systems; AI agent interactions | - Defines trustworthiness as the guarantee that agents will act as expected without supervision. - Operationalizes via ratio of successful-to-total actions compared against a predefined threshold; agents exceeding the threshold are deemed trustworthy. - Provides a transparent, mathematically simple mechanism for trustworthiness assessment. | - Requires extensive past performance data, problematic under nuclear data-sparse conditions (rare-event regimes). - Threshold determination is informal; not aligned with nuclear regulatory acceptance criteria. - No consideration of socio-technical operating context. |
| (Sun et al., 2016) | ML-based Prognostics and Health Management (PHM) | - Defines trustworthiness of prognostics (ToP) as the degree to which a prognostic system meets end-user requirements for a specific application. - Operationalizes ToP via Relative Accuracy (RA), Relative Precision (RP), Prognostic Horizon (PH), and resource occupancy. - Supports offline, online, and comprehensive assessment across verification and in-service settings. - Explicitly treats predictive uncertainty and differentiates accuracy, precision, and timeliness. | - Requires repeated prediction–observation datasets and run-to-failure histories, scarce in NPP contexts. - User-defined acceptance zones, confidence levels, and aggregation weights introduce subjectivity. - Multiplicative subsystem aggregation may over-penalize when one module underperforms. - No consideration of socio-technical operating context. |
| ***(A.2.1) Output Deviation-based – Implicit Definition – Nuclear Domain*** | | | |
| (Lin, Bao, et al., 2021); and (Lin, Lee, et al., 2021) | ML-based Digital Twin (DT) within the NAMAC framework for NPP operations | - Real-time discrepancy checker flags DT outputs as untrustworthy when deviation from plant/simulator observations exceeds a predefined limit (triggering a scram signal). - Integrates uncertainty quantification (UQ) with software risk analysis, aligning with NRC risk-informed model assessment. - Individual DTs achieve ≤5% L1 relative error within training domain; confusion matrices reveal degradation outside training domain, justifying the fail-safe checker. | - Distance metric and deviation threshold are left unspecified, limiting regulatory defensibility. - Performance is strongly dependent on training-domain coverage. - No explicit mapping between trustworthiness-loss thresholds and plant-level risk acceptance criteria (e.g., core damage frequency). - Limited consideration of socio-technical operating context. |
| (Hanna et al., 2021) | ML-based Operator Support System (NAMAC) in NPP control rooms | - Evaluates real-time 'trustability' of NAMAC via three discrepancy classes: (i) training-vs.-actual scenario dissimilarity; (ii) ML prediction vs. observed sensor values; (iii) expected vs. actual effect of corrective actions. - Uses Answer Set Programming (ASP) for online reasoning over logical trustability conditions. - Validated across 22 simulated Loss-of-Flow (LOF) scenarios; combined metrics detect non-trustable recommendations with high accuracy. | - Dissimilarity thresholds and action-delay tolerances are user-defined and may lack regulatory defensibility. - Uncertainty is not propagated across NAMAC modules. - Reliance on single-scenario training constrains generalization to diverse NPP transients. - Limited consideration of socio-technical operating context. |
| (Kim et al., 2021) | ML-based fire detection for nuclear-relevant facilities (validated on NIST/NRC test series) | - Computes reconstruction error (Max Squared Error, MaxSE) between real sensor inputs and encoder-decoder outputs; exceeding the threshold triggers a fallback to a knowledge-based rule method. - Hybrid fail-safe design explicitly handles out-of-distribution conditions. - Validated using NRC-sponsored fire safety experiments including nuisance scenarios (cooking, smoking) and sensor faults. | - Deviation threshold is empirically derived without regulator-anchored justification. - Synthetic (CFD-based) training data may not fully capture nuclear fire dynamics. - No explicit linkage between trustworthiness loss and risk-informed acceptance criteria (e.g., fire-induced core damage frequency). - Limited consideration of socio-technical operating context. |
| ***(A.2.2) Output Deviation-based – Implicit Definition – Non-Nuclear Domain*** | | | |
| (Chen et al., 2021)  (Rahmati et al., 2023) | PLC code integrity (industrial control) ML predictions for nano-lubricant tribology | - (Chen) Detects PLC code tampering by flagging deviations between live actuator outputs and neural-network-ensemble predictions; validated against testbed adversarial attacks. - (Rahmati) Frames trustworthiness as agreement between model-estimated and experimentally measured viscosities across a wide operating range; strong empirical validation. - Both studies demonstrate clear, quantifiable deviation metrics with strong domain-specific empirical validation. | - Thresholds are empirically set and not anchored to regulatory acceptance criteria. - Evaluation is constrained by available data and may fail in rare-event regimes relevant to nuclear safety. - No uncertainty quantification beyond performance statistics. - No consideration of socio-technical operating context. |

- Section A-3: Synthesis of Literature on the Definitions and Methodologies of AI-Automation Trustworthiness Using the Attribute-based approach

| **Study** | **Context** | **Key Insights** | **Limitations for Nuclear Deployment** |
| --- | --- | --- | --- |
| ***(B.1.1) Attribute-based – Explicit Definition – Nuclear Domain*** | | | |
| (Smidts et al., 2019) with attributes from (Avizienis et al., 2004) and (Sterbenz et al., 2010) | Networked Industrial Control Systems (ICS) | - Defines trustworthiness as a set of measurable properties for monitoring system performance over time. - Three trustworthiness components: (1) system dependability (reliability, availability, safety, integrity, maintainability); (2) system security (availability, integrity, confidentiality); (3) performability (quality-of-service measures). - Introduces mathematical models for real-time quantification of reliability and availability. | - Inherent subjectivity of some attributes (e.g., safety, maintainability) limits objective measurement. - No justification that the proposed attribute set is sufficient to fully characterize automation trustworthiness. - Aggregation of attributes into overall trustworthiness is unaddressed; linear and nonlinear interdependencies require formal validation. |
| (Wang, 2022) | Nearly Autonomous Management and Control (NAMAC) system for NPP operator support | - Defines trustworthiness as the assurance that NAMAC will work as expected. - Five attributes: reliability, explainability, safety, resilience, and security (with sub-attributes transparency and availability). - Develops a Bayesian Belief Network (BBN) framework using Conditional Probability Tables (CPTs) derived via expert elicitation to quantify trustworthiness during design and operational phases, one of the few studies providing a numeric trustworthiness estimate. | - CPTs derived via expert elicitation introduce subjective biases. - No consideration of socio-technical operating context (human-automation-physical interactions) in operational-phase evaluation. - No comparison of measured trustworthiness against predefined regulatory acceptance thresholds, essential for nuclear deployment decisions. |
| (Muhlheim et al., 2022) | Digital Twin (DT) for predictive maintenance in NPP | - Defines DT trustworthiness in terms of the quality, correctness, and fidelity of information presented to operators. - Emphasizes that potential risks to plant safety must be considered alongside trustworthiness evaluation. - Highlights the importance of regulatory review covering performance assessment, uncertainty determination, and DT transparency. | - No formal methodology proposed for evaluating or quantifying trustworthiness based on the three defined attributes. - Definition is descriptive only; no operationalization or acceptance thresholds provided. |
| (Saboury et al., 2023) | AI applications in nuclear medicine | - Defines trustworthiness as encompassing both technical AI attributes (reliability, predictability, robustness) and the integrity, competence, and benevolence of people and processes across the AI lifecycle. - Proposes a twelve-element framework: human agency, oversight, technical robustness, safety, accountability, data governance, diversity, fairness, stakeholder participation, transparency, privacy, sustainability, change control, and implementation context. | - No formal methodology to measure or quantify the twelve elements. - Framework is comprehensive in scope but lacks operationalization, no metrics or thresholds for deployment decisions. |
| (Wu et al., 2025) leveraging attributes from (Ali et al., 2023) | Verification, Validation, and Uncertainty Quantification (VVUQ) for ML models in nuclear applications | - Defines trustworthiness through four subdomains: explainability, interpretability, reproducibility, and applicability. - Proposes a VVUQ framework integrating these four attributes across the ML model lifecycle while explicitly accounting for uncertainties affecting model performance. - Ties trustworthiness to quality assurance processes as a pathway to regulatory confidence. | - Does not justify exclusion of other relevant trustworthiness attributes (e.g., safety, resilience). - No consideration of socio-technical operating context in the trustworthiness evaluation. - No comparison of measured trustworthiness against predefined regulatory acceptance thresholds. |
| ***(B.1.2) Attribute-based – Explicit Definition – Non-Nuclear Domain*** | | | |
| (Sheridan, 1989) and (Sheridan, 2019a) | Human factors in command-and-control systems | - (1989) Introduces seven attributes influencing user trust: reliability, robustness, understandability, explication of intent, usefulness, familiarity, and dependence. - (2019a) Refines framework by distinguishing trustworthiness (objective system property: reliability, robustness, understandability, explication of intent, usefulness) from trust (subjective user property: familiarity, dependence). - One of the earliest systematic attempts to formalize trustworthiness through well-defined system attributes. | - Proposed multiplicative combination of reliability, usefulness, and robustness as trustworthiness lacks empirical validation. - Operationalization of several attributes (e.g., understandability, explication of intent) remains ambiguous. - Framework is largely theoretical with no formal quantitative methodology. |
| (Grandison et al., 2000); later acknowledged by (Ugur, 2021) | Internet applications (medical information systems, information retrieval) | - Defines trust as the firm belief in an entity's competence to act dependably, securely, and reliably within a specified context (dependability assumed to cover reliability and timeliness). - One of the earliest formal frameworks for trust management in distributed systems. | - Conflation of 'trust' and 'trustworthiness' introduces conceptual ambiguity. - Security-centric attribute set does not capture broader socio-technical dimensions relevant to nuclear human-interactive systems. - No quantitative methodology for measuring trustworthiness. |
| (Smuha, 2019); later acknowledged by (Fernández Llorca et al., 2021) and (Borg et al., 2021). | European Commission AI policy, cross-domain | - Defines trustworthy AI through three core attributes: lawful, ethical, and robust. - Further specifies transparency, verifiability, explainability, security, technical robustness, and safety as necessary supporting properties. - Emphasizes contextual interpretation of attributes based on specific AI domain and application. | - No operational metrics or standardized methodologies for quantifying or validating these attributes in practice. - Policy-level framework not calibrated for safety-critical nuclear regulatory contexts. |
| (NIST, 2023) later applied by (Chen et al., 2024) | NIST AI Risk Management Framework, cross-domain | - Defines trustworthy AI through seven characteristics: valid and reliable, safe, secure and resilient, accountable and transparent, explainable and interpretable, privacy-enhanced, and fair with harmful bias managed. - Introduces a four-function operational core: Govern, Map, Measure, and Manage, to systematically address AI risks across the system lifecycle. - Applied by Chen et al. (2024) via the DARE method: a real-time data supervisor assessing whether test inputs are sufficiently supported by training data for reliable NPP model predictions. | - Limited quantitative guidance or standardized metrics for measuring the seven characteristics in practice. - Framework provides risk-management structure but does not specify how to determine whether achieved trustworthiness meets regulatory acceptance thresholds. |
| (Thiebes et al., 2021) | Information Systems (IS), AI lifecycle | - Defines Trustworthy AI (TAI) through five foundational principles: beneficence, non-maleficence, autonomy, justice, and explicability. - Proposes DaRe4TAI: a data-driven research framework bridging these principles to practical challenges across the AI lifecycle (data input, model development, output). - Strong theoretical integration of ethical and technical perspectives. | - DaRe4TAI is a conceptual research tool; not a trustworthiness evaluation method. - No formal methodology or case study for quantitatively measuring or operationalizing AI trustworthiness. |
| (Finkel et al., 2021) acknowledged the definition by XM Shi | AI in high-performance scientific computing | - Describes trustworthiness with five key components: security, privacy, fairness, provenance, and integrity, each explicitly defined and contextually explained. | - No quantitative methods for measuring the five components. - No methodology for aggregating component-level assessments into an overall trustworthiness evaluation. |
| (Schiegl et al., 2021) | Semantic models for natural language processing (NLP) | - Frames trustworthiness as users' perception of and confidence in model quality. - Links trustworthiness to quality dimensions: correctness, completeness, and relevancy. | - Perception-based definition introduces subjectivity and inter-user inconsistency. - No formalized methods for objectively measuring trustworthiness or its underlying quality dimensions. |
| (Czekster et al., 2021) | Cyber-Physical Systems (CPS) | - Frames trustworthiness in terms of security, privacy, safety, reliability, and resilience. - Emphasizes that CPS trustworthiness must be ensured by both managers and customers. | - No formal methodology or metrics proposed for evaluating the identified attributes. - Trustworthiness remains qualitative and interpretive without operationalization. |
| (Wittkotter et al., 2021) | Artificial Super Intelligence (ASI) security | - Frames trustworthiness around security: a trustworthy ASI must operate under strict security policies, resist exploitation, detect breaches, mitigate impact, and autonomously reduce downtime. - Highlights internal safeguards as a core trustworthiness mechanism in high-stakes autonomous systems. | - Does not specify who defines or validates the security policies and thresholds. - Security-centric framing is insufficient for safety-critical nuclear applications where trustworthiness must also encompass safety, reliability, and operational efficiency. |
| (Wang et al., 2021) | Physics-guided Deep Learning (DL) for dynamical systems | - Frames trustworthiness as AI model adherence to fundamental physical laws (e.g., thermodynamics, quantum mechanics). - Identifies generalizability, sustaining predictive accuracy across varying parameters, boundary conditions, and external forces, as an essential trustworthiness attribute. - Physical law embedding enables models to capture true system dynamics rather than fitting observed data. | - Scope confined to systems governed by well-defined physical equations. - Limited applicability in nuclear contexts where governing equations are highly coupled and complex, making direct physical constraint embedding difficult. - No quantitative framework for assessing overall trustworthiness. |
| (Alsaigh et al., 2022): with leveraging from (Phillips et al., 2020) and (Yigitcanlar et al., 2021) | AI for smart energy systems | - Defines trustworthiness using thirteen attributes: explainability, interpretability, accuracy, privacy, reliability, robustness, safety, security/resilience, bias mitigation, transparency, fairness, accountability, and responsibility. - Integrates ethical, technical, and governance dimensions for AI in critical infrastructure. | - No formal framework or quantitative methodology for measuring the attributes or evaluating overall trustworthiness. |
| (OECD, 2021); acknowledged by (Freeman et al., 2022) | OECD AI Principles, cross-domain policy | - Defines trustworthy AI as systems embodying OECD principles: human rights and privacy, fairness, transparency, explainability, robustness, security, safety, and accountability. - Extends principles into a comparative framework classifying tools and practices by lifecycle stage (technical, procedural, educational). | - No direct mechanism for evaluating or quantifying trustworthiness. - Policy-level framework not calibrated for safety-critical nuclear regulatory contexts. |
| (Zhou et al., 2023) | Deep Neural Networks (DNNs) for industrial fault diagnosis and prognostics | - Treats uncertainty estimation as a measurable trustworthiness attribute. - Integrates VGG network with Dempster-Shafer evidence theory, replacing the SoftMax layer with a Dirichlet-distribution output to simultaneously estimate belief mass and uncertainty. - Demonstrates inverse relationship: higher belief mass corresponds to lower uncertainty, enhancing interpretability and robustness in out-of-distribution (OOD) scenarios. - Validated on industrial datasets; improves diagnostic performance under uncertain conditions. | - No consideration of socio-technical operating context in trustworthiness evaluation. - No comparison of achieved trustworthiness against predefined regulatory acceptance thresholds. - No linkage to risk-informed safety criteria relevant to nuclear deployment. |
| (Ali et al., 2023); with cited insights from (Hall et al., 2024) and (Wu et al., 2025). | Explainable AI (XAI), healthcare, military, banking | - Identifies seven key requirements for Trustworthy AI: robustness and safety, human agency and oversight, transparency, privacy and data governance, diversity/non-discrimination/fairness, accountability, and societal and environmental well-being. - Positions explainability as a core enabler of trustworthiness, essential for regulatory compliance and user confidence in high-stakes applications. | - Provides conceptual understanding only, no formal methodology or empirical framework for measuring or operationalizing trustworthiness. - No quantitative aggregation model linking the seven requirements to an overall trustworthiness metric. |
| (Manchanda, 2022) | Data science and machine learning, lifecycle management | - Defines trustworthiness through eight attributes: reliability, explainability, safety, privacy, responsibility, impartiality, sustainability, and human-centric design. - Proposes an End-to-End Agile and Automated ML Framework embedding quality-assurance checkpoints, automated data validation, bias detection, and continuous performance monitoring throughout the model lifecycle. | - Does not clearly distinguish environmental/contextual factors (e.g., varying lighting) from system attributes (e.g., explainability) in the trustworthiness model. - Benchmark for evaluating trustworthiness improvement is not formally defined or justified. - No consideration of socio-technical operating context across lifecycle phases. |
| (Roszel, 2024) | Privacy-preserving collaborative machine learning | - Identifies six trustworthiness principles: accountability, explainability and interpretability, fairness and non-discrimination, privacy, robustness and reliability, and safety and security. - Proposes the Know Your Model (KYM) framework: distills six principles into four concepts (efficacy, reliability, safety, responsibility) with lifecycle-wide qualitative guidelines for transparency and regulatory compliance. | - No justification for why the six principles represent a sufficient set. - KYM framework does not offer quantitative guidance for assessing how attributes individually or collectively affect overall trustworthiness. - No mechanism for determining whether achieved trustworthiness is sufficient for a given application context. |
| (Franco, 2024) | Supervised ML for cybersecurity and reliable AI | - Defines trustworthiness through four attributes: explainability, fairness, privacy, and robustness. - Develops algorithms and frameworks delivering privacy (Homomorphic Encryption), fairness (fair data representations), and explainability (local/global explanation techniques) by design. - Proposes a theoretical framework for robustness-bias mitigation under fairness and robustness constraints simultaneously. | - Lacked justification for the appropriateness of attribute set. - Heterogeneity of ML models and application contexts complicates consistent operationalization. - No quantitative metric linking attributes to overall trustworthiness or to regulatory deployment thresholds. |
| ***(B.2.1) Attribute-based – Implicit Definition – Nuclear Domain*** | | | |
| (OHara et al., 2010) | Human-system interface (HSI) design guidance for NPP automation | - Identifies reliability as a key trustworthiness attribute: operators may deem a system untrustworthy if it frequently produces false alarms or inaccurate guidance. | - Single-attribute (reliability) perspective, no discussion of other trustworthiness dimensions. - No formal definition, methodology, or quantitative framework for evaluating trustworthiness. |
| (Tantawy et al., 2019) | Digital Instrumentation and Control (I&C) systems for NPP | - Characterizes trustworthy digital I&Cs as systems that meet user and operator expectations even under challenging conditions: disruptions, faults, operator errors, vulnerabilities, and cyber-attacks. - Treats 'proper functioning under adversity' as synonymous with trustworthiness. | - No explicit definition of trustworthiness or its constituent attributes. - No formal methodology or quantitative framework for evaluating trustworthiness. |
| (Agarwal et al., 2022) | Technical basis for advanced AI/ML adoption in NPP | - Identifies efficiency, reliability, explainability, and accuracy as key trustworthiness-influencing attributes. - Positions explainability as an essential attribute for enhancing trust in AI. - Notes that AI need not be comprehensible to be trustworthy if it can be proven to perform accurately (citing Kozyrkov, 2022). | - Attribute set is not justified as comprehensive. - No methodology to measure, validate, or assess the collective influence of attributes on overall trustworthiness. - No formal definition of trustworthiness provided. |
| (Kollias et al., 2022) | ML-based noise diagnostics for anomaly detection in nuclear reactor cores | - Argues that trust in ML predictions is fostered by incorporating uncertainty quantification (UQ), confidence estimation, and enhancing interpretability and explainability of model outputs. | - Does not clarify whether the identified attributes (UQ, interpretability, explainability) comprehensively capture all trustworthiness dimensions. - No methodology to measure or validate these attributes collectively. - No formal definition of trustworthiness provided. |
| (Kobayashi et al., 2023) | AI-driven Remaining Useful Life (RUL) prediction for NPP components | - Operationalizes trustworthiness through five attributes: accuracy, overfitting, reliability, robustness, and resilience. - Uses distinct quantitative techniques per attribute: MSE/MAE/R2 (accuracy); training-vs-test comparison (overfitting); split conformal prediction and distribution-shift testing (reliability); covariate perturbation (robustness); worst-case subsampling (resilience). - Compares four AI algorithms (Tree, EBM, Figs, ReLU-DNN) to identify the most trustworthy under diverse operational conditions. | - No unified framework aggregating five attributes into an overall trustworthiness metric. - Does not establish whether the achieved trustworthiness level is adequate for nuclear deployment. - Attribute selection not justified as comprehensive and no guidance was proposed on the selection of attributes for a range of contexts and applications. |
| (Alberti et al., 2023) | AI-driven automation-level selection for nuclear reactor operations | - Asserts that AI trustworthiness is realized through explainability, approached in terms of: algorithmic fairness, identification of data biases, expected algorithm performance, and rational explanations for decisions. | - Reduces trustworthiness to a single attribute (explainability) without justification. - No formal linkage between explainability factors and overall trustworthiness. - No methodology to measure or validate trustworthiness. |
| (Walker et al., 2023) later extended by (Lin et al., 2025). | Explainable AI for predictive maintenance under the LWRS Program | - Conceptualizes AI trustworthiness through three approaches: (1) trust-but-verify (linking human validation to system trust); (2) data novelty detection (identifying distributional shifts); (3) value of new information (enabling system to request additional data to refine outputs). - Positions explainability and trustworthiness as coequal and interdependent, unlike most works where explainability is a sub-attribute. - Extended by Lin et al. (2025) using post-hoc explainability methods (SHAP, LIME) for circulating water system predictive maintenance. | - Subjectivity of the 'trust-but-verify' approach complicates objective trustworthiness measurement. - No formal framework linking the three approaches to an overall trustworthiness metric. |
| (Mortenson et al., 2024) building on (Shneiderman, 2020) | Human factors for AI applications in NPP | - Frames AI trustworthiness as achieved through governance: structured, independent, multilayered public-private oversight mechanisms. - In nuclear contexts, oversight contributors include EPRI, NEI, NRC, IAEA, IEEE, and ASME. | - No quantitative framework for evaluating trustworthiness. - Explainability and interpretability are listed as trustworthiness components alongside governance, conflating process and attribute perspectives. |
| ***(B.2.2) Attribute-based – Implicit Definition – Non-Nuclear Domain*** | | | |
| (Brust et al., 2020; Mandal et al., 2021; Sifakis, 2019) | Autonomous systems AI-driven groundwater classification Smart city AI systems | - (Sifakis) Characterizes trustworthiness via resilience to software flaws, hardware failures, human errors, and environmental disturbances; proposes a 0-1 trustworthiness scale where threshold is set by task criticality. - (Mandal) Defines trustworthiness as model's ability to generalize to unseen data; validated via ROC/AUC on held-out test data. - (Brust) Characterizes trustworthiness as encompassing data diversity, privacy preservation, and interconnected big-data properties. | - (Sifakis) Does not specify how to operationalize the 0-1 scale or determine criticality thresholds across diverse socio-technical contexts. - (Mandal, Brust) No consideration of socio-technical context; no formal guidance for determining sufficiency of achieved trustworthiness. - All three: no quantitative aggregation framework; no regulatory anchoring. |
| (Henderson et al., 2022; Shneiderman, 2020) | Human-Centered AI (HCAI) Aerospace engineering / airworthiness | - (Shneiderman) Emphasizes that trustworthy systems must be governed by respected oversight entities (ISO, IEEE, FDA, FAA) providing independent evaluation of design, operation, and maintenance, a view also cited by (Hall & Agarwal, 2024). - (Henderson) Equates trustworthiness with airworthiness: adherence to established certification standards (MIL-HDBK-516C, DO-178C, FAA regulations), verification, data management, robustness, and collaborative control. | - Absence of a common vocabulary across multidisciplinary oversight entities. - No quantitative methodology for evaluating trustworthiness beyond compliance checklists. |
| (Buluc et al., 2021; Karra et al., 2021; Mahdavifar, 2021; Volkova et al., 2022) | Physics-informed ML for porous media Cybersecurity deep learning Scientific computing ML AI-based smart grid services | - (Karra) Identifies explainability, transparency, and interpretability as key trustworthiness features for physics-informed ML. - (Mahdavifar) Argues explainable DNNs enhance trustworthiness by making system behavior transparent. - (Buluc) Requires ML models to provide interpretable outputs with quantified sensitivity and uncertainty. - (Volkova) Associates accountability (closely tied to transparency) with enhanced trust in smart grid AI services. | - Recurring conflation of explainability, interpretability, and transparency, terms used interchangeably without clear distinction. - No formal definitions or methodologies for evaluating trustworthiness; attribute identification without operationalization. - All four: single-attribute focus without a unifying trustworthiness framework. |
| (Shore et al., 2021) (Ahmed et al., 2021; Findrik et al., 2017; Fisher et al., 2021) | Information technology (BS 10754-1) Autonomous inspection robotics Intrusion detection for nuclear facilities Reproducible machine learning | - (Shore) Outlines safety, reliability, availability, resilience, and security as five pillars of trustworthy IT (per BS 10754-1). - (Fisher) Identifies reliability as central to trustworthiness in autonomous robotics. - (Findrik) Links high false positive/negative rates in intrusion detection to reduced trustworthiness, directly relevant to NPP cybersecurity. - (Ahmed) Demonstrates trustworthiness through reproducibility: consistent outcomes under similar conditions by controlling random parameter initialization. | - No formal definitions or unified quantitative framework across these studies. - No consideration of socio-technical operating context or regulatory acceptance thresholds. - Attribute-level insights without methods for aggregating into overall trustworthiness or deployment justification. |
| (Ersöz et al., 2022; Zafer et al., 2024) | Trustworthy/Explainable AI in renewable energy Ethical and safe AI | - (Ersoz) Identifies six trustworthy AI dimensions: value congruence, justice, accountability, repeatability, explainability, and robustness, grouped into social (justice, accountability, value congruence) and technical (robustness, repeatability, explainability) categories. - (Zafer & Sani) Defines trustworthy AI as operating ethically and safely, integrating fairness, transparency, accountability, privacy, and safety throughout the AI lifecycle from development to deployment. | - Broad ethical framing not operationalized with quantitative metrics or regulatory acceptance criteria. - No consideration of socio-technical operating context specific to safety-critical domains. - No mechanism for determining whether achieved trustworthiness meets deployment thresholds. |

**REFERENCES**

Idaho National Laboratory (INL), Idaho Falls, ID (United States). (2022). *Technical basis for advanced artificial intelligence and machine learning adoption in nuclear power plants*.

Sandia National Lab.(SNL-NM), Albuquerque, NM (United States). (2021). *Quantifying the Crisis in Reproducible Machine Learning*.

Alberti, A. L., Agarwal, V., Gutowska, I., Palmer, C. J., & de Oliveira, C. R. (2023). Automation levels for nuclear reactor operations: A revised perspective. *Progress in Nuclear Energy*, *157*, 104559.

Ali, S., Abuhmed, T., El-Sappagh, S., Muhammad, K., Alonso-Moral, J. M., Confalonieri, R., Guidotti, R., Del Ser, J., Díaz-Rodríguez, N., & Herrera, F. (2023). Explainable Artificial Intelligence (XAI): What we know and what is left to attain Trustworthy Artificial Intelligence. *Information fusion*, *99*, 101805.

Alsaigh, R., Mehmood, R., & Katib, I. (2022). AI Explainability and Governance in Smart Energy Systems: A Review. *arXiv preprint arXiv:2211.00069*.

Avizienis, A., Laprie, J.-C., Randell, B., & Landwehr, C. (2004). Basic concepts and taxonomy of dependable and secure computing. *IEEE transactions on dependable and secure computing*, *1*(1), 11-33.

Borg, M., Bronson, J., Christensson, L., Olsson, F., Lennartsson, O., Sonnsjö, E., Ebabi, H., & Karsberg, M. (2021). Exploring the assessment list for trustworthy ai in the context of advanced driver-assistance systems. 2021 IEEE/ACM 2nd International Workshop on Ethics in Software Engineering Research and Practice (SEthics),

Brust, M. R., Bouvry, P., Danoy, G., & Talbi, E.-G. (2020). Design Challenges of Trustworthy Artificial Intelligence Learning Systems. Asian Conference on Intelligent Information and Database Systems,

Buluc, A., Kolda, T. G., Wild, S. M., Anitescu, M., DeGennaro, A., Jakeman, J., Kamath, C., Kannan, R., Lopes, M. E., & Martinsson, P.-G. (2021). Randomized algorithms for scientific computing (RASC). *arXiv preprint arXiv:2104.11079*.

Chen, E., Bao, H., & Dinh, N. (2024). Evaluating the reliability of machine-learning-based predictions used in nuclear power plant instrumentation and control systems. *Reliability Engineering & System Safety*, *250*, 110266.

Chen, Y., Poskitt, C. M., & Sun, J. (2021). Code integrity attestation for PLCs using black box neural network predictions. Proceedings of the 29th ACM Joint Meeting on European Software Engineering Conference and Symposium on the Foundations of Software Engineering,

Czekster, R. M., Morisset, C., Clark, J. A., Soudjani, S., Patsios, C., & Davison, P. (2021). Systematic review of features for co‐simulating security incidents in cyber‐physical systems. *Security and Privacy*, *4*(3), e150.

Ersöz, B., Sağıroğlu, Ş., & Bülbül, H. (2022). Methods of Explainable Artificial Intelligence (XAI), Trustworthy Artificial Intelligence (TAI) and Interpretable Machine Learning (IML) in Renewable Energy. *International Journal of Smart Grid-ijSmartGrid*, *6*(4).

Fernández Llorca, D., & Gómez, E. (2021). Trustworthy autonomous vehicles. *Publications Office of the European Union, Luxembourg,, EUR*, *30942*.

Findrik, M., Friedberg, I., Piatkowska, E., Smith, P., & Song, J.-G. (2017). Trustworthy computer security incident response for nuclear facilities. Proc. of the 4th International Symposium on Future Instrumentation and Control for Nuclear Power Plants (ISOFIC’17), Gyeongju, South Korea,

US Department of Energy (USDOE), Washington DC (United States). Office of …. (2021). *Responses to the Request for Information on Stewardship of Software for Scientific and High-Performance Computing*.

Fisher, M., Cardoso, R. C., Collins, E. C., Dadswell, C., Dennis, L. A., Dixon, C., Farrell, M., Ferrando, A., Huang, X., Jump, M., Kourtis, G., Lisitsa, A., Luckcuck, M., Luo, S., Page, V., Papacchini, F., & Webster, M. (2021). An Overview of Verification and Validation Challenges for Inspection Robots. *Robotics*, *10*(2). <https://doi.org/10.3390/robotics10020067>

Franco, D. (2024). Towards Trustworthiness in Artificial Intelligence: Pushing for Explainable, Fair, Robust, and Private Supervised Machine Learning.

Freeman, L., Batarseh, F. A., Kuhn, D. R., Raunak, M. S., & Kacker, R. N. (2022). The path to a consensus on artificial intelligence assurance. *Computer*, *55*(3), 82-86.

Grandison, T., & Sloman, M. (2000). A survey of trust in internet applications. *IEEE Communications Surveys & Tutorials*, *3*(4), 2-16.

Hall, A., & Agarwal, V. (2024). Barriers to adopting artificial intelligence and machine learning technologies in nuclear power. *Progress in Nuclear Energy*, *175*, 105295.

Hanna, B., Lin, L., Athe, P., Son, T., & Dinh, N. (2021). Trusting Machine Learning in Nuclear Plant Control: A Reasoning-Based Discrepancy Checker. 12th Nuclear Plant Instrumentation, Control and Human-Machine Interface Technologies,

Henderson, A., Harbour, S., & Cohen, K. (2022). Toward airworthiness certification for artificial intelligence (AI) in aerospace systems. 2022 IEEE/AIAA 41st Digital Avionics Systems Conference (DASC),

Karra, S., Ahmmed, B., & Mudunuru, M. K. (2021). AdjointNet: Constraining machine learning models with physics-based codes. *arXiv preprint arXiv:2109.03956*.

Kim, Y.-J., Kim, H., Lee, S., & Kim, W.-T. (2021). Trustworthy building fire detection framework with simulation-based learning. *IEEE Access*, *9*, 55777-55789.

Kobayashi, K., Almutairi, B., Sakib, M. N., Chakraborty, S., & Alam, S. B. (2023). Explainable, Interpretable & Trustworthy AI for Intelligent Digital Twin: Case Study on Remaining Useful Life. *arXiv preprint arXiv:2301.06676*.

Kollias, S., Yu, M., Wingate, J., Durrant, A., Leontidis, G., Alexandridis, G., Stafylopatis, A., Mylonakis, A., Vinai, P., & Demaziere, C. (2022). Machine learning for analysis of real nuclear plant data in the frequency domain. *Annals of Nuclear Energy*, *177*, 109293.

Lin, L., Bao, H., & Dinh, N. (2021). Uncertainty quantification and software risk analysis for digital twins in the nearly autonomous management and control systems: A review. *Annals of Nuclear Energy*, *160*. <https://doi.org/10.1016/j.anucene.2021.108362>

Lin, L., Lee, J., Poudel, B., McJunkin, T., Dinh, N., & Agarwal, V. (2021). Enhancing the operational resilience of advanced reactors with digital twins by recurrent neural networks. 2021 Resilience Week (RWS),

Lin, L., Walker, C., & Agarwal, V. (2025). Explainable machine-learning tools for predictive maintenance of circulating water systems in nuclear power plants. *Nuclear Engineering and Technology*, *57*(9), 103588.

Ma, H., Liu, L., Luo, H., Ma, H., Liu, L., & Luo, H. (2021). Data Fusion Based Transmission in Multimedia Sensor Networks. *Multimedia Sensor Networks*, 85-143.

Mahdavifar, S. (2021). Explainable deep learning for detecting cyber threats.

Manchanda, S. (2022). End to End Agile and Automated Machine Learning Framework for Trustworthy, Reliable and Sustainable Artificial Intelligence. International Conference on Data Management, Analytics & Innovation,

Mandal, P., Saha, J., Bhattacharya, S., & Paul, S. (2021). Delineation of groundwater potential zones using the integration of geospatial and MIF techniques: A case study on Rarh region of West Bengal, India. *Environmental Challenges*, *5*, 100396.

Mortenson, T., Kovesdi, C., & Mohon, J. (2024). Human Factors Considerations in Artificial Intelligence Applications for Nuclear Power Plants. *Nuclear Technology*, 1-17.

Oak Ridge National Lab.(ORNL), Oak Ridge, TN (United States). (2022). *How Digital Twins May Be Used in Design and Operations*.

National Institute of Standards and Technology, U.S. Department of Commerce. (2023). *Artificial Intelligence Risk Management Framework (AI RMF 1.0)* (NIST AI 100-1). <https://doi.org/10.6028/NIST.AI.100-1>

OECD. (2021). Tools for trustworthy AI. <https://doi.org/doi:https://doi.org/10.1787/008232ec-en>

OHara, J. M., & Higgins, J. (2010). Human-system interfaces to automatic systems: Review guidance and technical basis.

Phillips, P. J., Hahn, C. A., Fontana, P. C., Broniatowski, D. A., & Przybocki, M. A. (2020). Four principles of explainable artificial intelligence. *Gaithersburg, Maryland*, 18.

Rahmati, M., Tanha, A. A., & Abolfazli, S. K. (2023). A new empirical model for accurate investigation of rheological behavior of MWCNT/oxide nanoparticles-engine oil hybrid nano-lubricants. *Tribology International*, *182*, 108337.

Roszel, M. (2024). Towards Trustworthy Artificial Intelligence in Privacy-Preserving Collaborative Machine Learning.

Saboury, B., Bradshaw, T., Boellaard, R., Buvat, I., Dutta, J., Hatt, M., Jha, A. K., Li, Q., Liu, C., & McMeekin, H. (2023). Artificial intelligence in nuclear medicine: opportunities, challenges, and responsibilities toward a trustworthy ecosystem. *Journal of Nuclear Medicine*, *64*(2), 188-196.

Schiegl, M., Hörfarter, C., Novak, M., Kondrová, L., Hernández, R., Pabón, M. S., & van Ede, R. (2021). *GeoERA Final Report on Keyword Thesaurus and Project Vocabularies-Experiences and Status of the Work*. Geologische Bundesanstalt.

Schmid, S., Riebe, T., & Reuter, C. (2022). Dual-Use and Trustworthy? A Mixed Methods Analysis of AI Diffusion Between Civilian and Defense R&D. *Science and Engineering Ethics*, *28*(2), 12. <https://doi.org/10.1007/s11948-022-00364-7>

Sheridan, T. (1989). Trustworthiness of command and control systems. In *Analysis, Design and Evaluation of Man–Machine Systems 1988* (pp. 427-431). Elsevier.

Sheridan, T. B. (2019a). Individual differences in attributes of trust in automation: measurement and application to system design. *Frontiers in Psychology*, *10*, 1117.

Shneiderman, B. (2020). Human-centered artificial intelligence: Reliable, safe & trustworthy. *International Journal of Human–Computer Interaction*, *36*(6), 495-504.

Shore, M., Zeadally, S., & Keshariya, A. (2021). Zero trust: the what, how, why, and when. *Computer*, *54*(11), 26-35.

Sifakis, J. (2019). Can we trust autonomous systems? Boundaries and risks. Automated Technology for Verification and Analysis: 17th International Symposium, ATVA 2019, Taipei, Taiwan, October 28–31, 2019, Proceedings 17,

Smidts, C., Diao, X., & Vaddi, P. K. (2019). Next-Generation Architecture and Autonomous Cyber-Defense. *Industrial Control Systems Security and Resiliency: Practice and Theory*, 203-234.

Smuha, N. A. (2019). The EU approach to ethics guidelines for trustworthy artificial intelligence. *Computer Law Review International*, *20*(4), 97-106.

Sterbenz, J. P., Hutchison, D., Çetinkaya, E. K., Jabbar, A., Rohrer, J. P., Schöller, M., & Smith, P. (2010). Resilience and survivability in communication networks: Strategies, principles, and survey of disciplines. *Computer networks*, *54*(8), 1245-1265.

Sun, B., Jiang, X., Ye, T., & Feng, Q. (2016). A novel concept and assessment method for trustworthiness of prognostics. *Advances in Mechanical Engineering*, *8*(3), 1687814016638807.

Taddeo, M. (2010). Modelling trust in artificial agents, a first step toward the analysis of e-trust. *Minds and machines*, *20*, 243-257.

(2019). *Realizing Verifiable I&C and Embedded Digital Devices for Nuclear Power* (DE-NE0008445). <https://www.researchgate.net/publication/343016814_Realizing_Verifiable_IC_and_Embedded_Digital_Devices_for_Nuclear_Power_Design_Verification_and_Demonstration_of_the_SymPLe_Architecture>

Thiebes, S., Lins, S., & Sunyaev, A. (2021). Trustworthy artificial intelligence. *Electronic Markets*, *31*, 447-464.

Ugur, A. (2021). Manipulator: A novel collusion attack on trust management systems in social IoT. Software Engineering and Algorithms: Proceedings of 10th Computer Science On-line Conference 2021, Vol. 1,

Volkova, A., Patil, A. D., Javadi, S. A., & de Meer, H. (2022). Accountability challenges of AI in smart grid services. Proceedings of the Thirteenth ACM International Conference on Future Energy Systems,

Idaho National Laboratory (INL), Idaho Falls, ID (United States). (2023). *Explainable artificial intelligence technology for predictive maintenance*.

Wang, L. (2022). Development and Application of Data Coverage Assessment for NAMAC Trustworthiness.

Wang, R., & Yu, R. (2021). Physics-guided deep learning for dynamical systems: A survey. *arXiv preprint arXiv:2107.01272*.

Wittkotter, E., & Yampolskiy, R. (2021). *Kill-Switch for Artificial Superintelligence*. ASI Safety Lab Inc. <https://asi-safety-lab.com/DL/Kill-Switch-For_ASI_EW_21_12_14.pdf>

Wu, X., Moloko, L. E., Bokov, P. M., Delipei, G. K., Kaizer, J., & Ivanov, K. N. (2025). Uncertainty Quantification for Data-Driven Machine Learning Models in Nuclear Engineering Applications: Where We Are and What Do We Need? *arXiv preprint arXiv:2503.17385*.

Yigitcanlar, T., Corchado, J. M., Mehmood, R., Li, R. Y. M., Mossberger, K., & Desouza, K. (2021). Responsible urban innovation with local government artificial intelligence (AI): A conceptual framework and research agenda. *Journal of Open Innovation: Technology, Market, and Complexity*, *7*(1), 71.

Zafer, M., & Sani, M. (2024). Trustworthy Artificial Intelligence: Fine-Tuning Machine Learning Models with Ethics and Safety.

Zhou, H., Chen, W., Cheng, L., Liu, J., & Xia, M. (2023). Trustworthy Fault Diagnosis with Uncertainty Estimation through Evidential Convolutional Neural Networks. *IEEE Transactions on Industrial Informatics*.
